# A Systematic Taxonomy of the Sunflower Optimization Algorithm: Variants, Hybridization Strategies, Applications, and Research Directions

**DOI:** 10.3390/biomimetics11060439

**Published:** 2026-06-20

**Authors:** Ceren Baştemur Kaya

**Affiliations:** Department of Computer Technologies, Nevşehir Vocational School, Nevşehir Hacı Bektaş Veli University, Nevşehir 50100, Türkiye; ceren@nevsehir.edu.tr or cerenbastemurkaya@gmail.com

**Keywords:** bio-inspired algorithm, sunflower optimization, metaheuristic algorithm, artificial intelligence

## Abstract

Due to the rapidly increasing number of studies conducted using SFO in recent years, a comprehensive and systematic review of the existing literature has become necessary. SFO is a bio-inspired metaheuristic optimization algorithm developed based on the sun-tracking behavior of sunflower plants. Owing to its simple mathematical structure and flexible search capability, SFO has been increasingly applied to various engineering and AI problems. This review study presents a systematic and comprehensive analysis of SFO-based studies published in the literature. The literature search was performed using the Scopus database, and a total of 192 studies were included in the final evaluation process. The reviewed studies were classified into eight major application domains, including engineering design, energy systems, machine learning, image processing, communication systems, robotics, forecasting, and multi-objective optimization. In addition, the distributions of standard, hybrid, and modified SFO approaches were comparatively analyzed. The temporal evolution of SFO studies, hybridization tendencies, application diversity, strengths, limitations, and future research directions were also systematically evaluated. The findings indicate that hybrid and modified SFO structures have become increasingly dominant in recent years, particularly in AI and data-driven optimization applications. Overall, this review provides a broad understanding of the current state and future research potential of SFO-based optimization studies.

## 1. Introduction

Metaheuristic optimization algorithms have become one of the most important solution approaches for complex engineering and AI problems in recent years [1,2,3,4]. These algorithms are widely preferred because they can effectively handle nonlinear, multi-dimensional, and highly constrained optimization problems without requiring gradient information [1,2,3,5]. In addition, the increasing complexity of modern optimization problems in engineering, communication systems, energy management, machine learning, and intelligent control applications has significantly increased the research interest in nature-inspired optimization methods [1,2,5,6].

Nature-inspired optimization algorithms are generally developed by modeling biological, physical, or social behaviors observed in real-world systems [1,2,4]. Nature-inspired and evolutionary optimization approaches, in particular, have attracted considerable attention due to their strong exploration and exploitation capabilities [1,5,6]. Over the years, many optimization algorithms inspired by natural systems have been proposed to improve convergence performance, optimization accuracy, computational efficiency, and solution diversity for different application domains [1,2,3,4,5,6,7].

Recent years have also witnessed the introduction of numerous new nature-inspired optimization algorithms, such as PFA, reflecting the continuing research interest in developing more effective exploration–exploitation strategies for complex optimization problems [8]. At the same time, the application scope of metaheuristic optimization algorithms has expanded considerably beyond traditional optimization tasks. These algorithms are increasingly being integrated with artificial intelligence techniques for neural network training, neuro-fuzzy system optimization, feature selection, parameter estimation, and forecasting applications. Such studies have demonstrated the effectiveness of metaheuristic algorithms in improving prediction accuracy, model performance, and optimization capability in complex nonlinear systems [9,10,11]. This growing interaction between optimization and artificial intelligence has further contributed to the development and widespread adoption of nature-inspired algorithms, including SFO.

Among these approaches, SFO algorithm has emerged as a relatively recent nature-inspired optimization method based on the sun-tracking behavior of sunflower plants [12]. The algorithm models the orientation and pollination mechanisms of sunflowers to guide the search process toward optimal solutions. Due to its relatively simple mathematical structure and flexible search capability, SFO has been increasingly applied to various optimization problems in recent years [13,14,15,16,17]. The reviewed literature shows that SFO has been successfully used in structural optimization, energy systems, communication networks, image processing, robotics, forecasting, machine learning, and intelligent optimization applications.

Another important trend observed in recent SFO studies is the increasing development of hybrid and modified optimization structures [18,19,20,21,22]. Researchers have increasingly combined SFO with machine learning frameworks, DL models, feature selection techniques, and other metaheuristic algorithms to improve optimization quality and convergence performance for more complex engineering problems. Particularly in image processing, forecasting, IoT systems, and prediction-oriented applications, hybrid SFO approaches have become more dominant than standard algorithm structures.

Despite the increasing number of SFO-based studies in the literature, a comprehensive and systematic review covering the application domains, hybridization trends, modified structures, temporal evolution, and research directions of SFO is still limited. Existing studies generally focus on specific application problems or individual optimization performances, while a broad analytical evaluation of SFO research trends across different domains remains insufficient.

Based on the reviewed literature, no comprehensive review study was identified that systematically examines SFO in terms of its application domains, standard, modified, and hybrid variants, hybridization strategies, research trends, strengths, limitations, and future research directions. Existing studies mainly focus on developing new methods and applying SFO to specific optimization problems. As a result, there is a need for a systematic review that provides a broader perspective on the overall development of SFO, its application diversity, and the evolution of its variants. To address this gap, the present study classifies SFO-related research into eight major application domains and provides a comprehensive analysis of the algorithm’s development, hybridization tendencies, research trends, and future research opportunities.

Therefore, this review study presents a comprehensive and systematic analysis of SFO-based studies published in the literature. The reviewed studies were classified into eight major application domains, and the distributions of standard, hybrid, and modified SFO approaches were comparatively evaluated. In addition, this study investigates the temporal evolution of SFO research, hybridization tendencies, application diversity, current limitations, and future research directions.

To guide the review process and provide a structured evaluation of the literature, the following research questions (RQ) were considered:

RQ1. What are the major application domains in which SFO has been applied?
RQ2. How have standard, modified, and hybrid SFO approaches evolved across different research domains?
RQ3. What strengths, limitations, and practical considerations can be identified from the existing SFO literature?
RQ4. What research gaps, open challenges, and future research directions emerge from current SFO studies?

The main contributions of this review can be summarized as follows:

- A comprehensive taxonomy of SFO application areas was developed based on eight major research domains.
- The distributions of standard, hybrid, and modified SFO variants were comparatively analyzed across different application areas.
- The temporal evolution and research trends of SFO studies were systematically evaluated.
- Hybridization tendencies and modified optimization structures in SFO-based studies were critically examined.
- The strengths, limitations, practical considerations, and future research opportunities of SFO were comprehensively discussed.

The findings of this review aim to provide a broad understanding of the current state and future potential of SFO for researchers working in optimization, engineering, and AI applications.

## 2. Background

### Sunflower Optimization Algorithm (SFO)

SFO is a nature-inspired optimization algorithm developed based on the sun-tracking behavior of sunflowers [12]. In this algorithm, each individual in the population represents a sunflower. The best solution in the population is considered as the “sun,” and the other individuals move according to this solution.

The algorithm combines orientation behavior and pollination mechanism within its structure. Through the interaction between individuals, new candidate solutions are generated while population diversity is maintained.

Another concept used in the SFO algorithm is the inverse square law. According to this principle, the radiation intensity is inversely proportional to the square of the distance. Therefore, individuals closer to the sun move with smaller steps, while individuals farther away perform larger movements.

The amount of energy received by each individual is calculated using the following equation:

(1) $$Q_{i}\;=\;\frac{P}{4\pi r_{i}^{2}}$$

Here, P represents the source power, while $r_{i}$ denotes the distance between the best individual and the related individual.

The orientation direction of the individuals toward the sun is determined by the following equation:

(2) $$\vec{s_{i}}\;=\;\frac{X^{*}\;-\;X_{i}}{\|X^{*}\;-\;X_{i}\|},\;i=\;1,\;2,\;\ldots ,\;n_{p}$$

The movement amount is calculated using the following equation:

(3) $$d_{i}=\lambda \times P_{i}(\|X_{i}+X_{i-1}\|)\times \;\|X_{i}+X_{i-1}\|$$

Here, λ represents a constant value that defines the “inertial” movement of the plants, while $P_{i}$($\|X_{i}+X_{i-1}\|$) represents the pollination probability.

In the algorithm, a maximum step limit is used to prevent individuals from making excessively large movements. The maximum movement amount is expressed as follows:

(4) $$d_{max}=\frac{\left\|X_{max}\;-\;X_{min}\right\|}{2\;\times \;N_{pop}}$$

Here, $X_{max}$ and $X_{min}$ represent the upper and lower bounds of the search space, while $N_{pop}$ denotes the total number of individuals in the population.

The new position of the individual is obtained using the following update equation:

(5) $${\vec{X}}_{i+1}={\vec{X}}_{i}+d_{i}\times \;{\vec{s}}_{i}$$

The algorithm starts with the generation of an initial population. Then, the best individual is identified and the remaining individuals are directed toward this solution. In each iteration, the orientation vectors and movement amounts are recalculated, new individuals are generated, and the population is updated. The process continues until the predefined maximum number of iterations is reached.

## 3. Review Methodology

In this review study, a comprehensive literature search process was conducted to systematically examine studies related to SFO. The literature search was performed only using the Scopus database. Scopus was selected as the main data source because it provides broad and reliable coverage of research in engineering, computer science, artificial intelligence, optimization, and applied mathematics. It also indexes both journal articles and conference papers, which is important for capturing recent developments in metaheuristic optimization. In addition, its advanced search tools, citation indexing, and standardized bibliographic records support a systematic and reproducible review process. For these reasons, Scopus was considered an appropriate source for identifying the main developments and research trends related to SFO.

The search query used in this study was defined as follows:

TITLE-ABS-KEY(

“sunflower optimization algorithm”

OR “sunflower optimization”

OR “sunflower algorithm”

OR “sunflower optimizer”

)

The literature search was conducted on 7 March 2026. In the initial search, a total of 237 studies were identified. After applying the document type filter, only journal articles and conference papers were included in the review, and the number of studies decreased to 223. All retrieved studies were found to be written in English; therefore, no additional language-based exclusion was required.

Afterward, the retrieved studies were examined in detail, and studies that did not directly focus on the SFO algorithm or only mentioned it superficially were excluded from the dataset. In addition, review papers, editorials, books, book chapters, short notes, and other non-research document types were excluded from the study. As a result of this screening process, the final number of included studies was reduced to 192.

**Figure 1:**
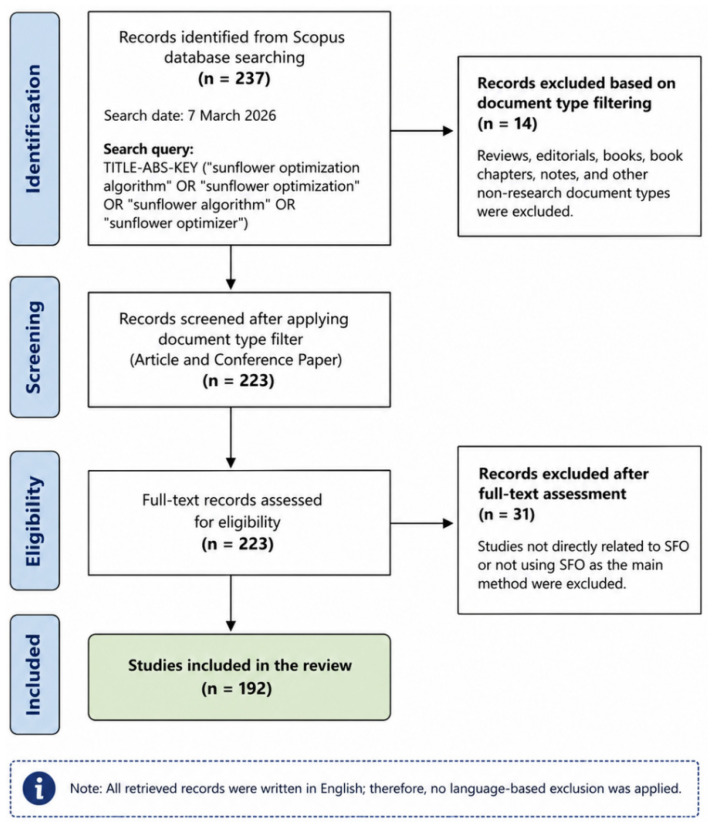
PRISMA flow diagram of the study selection process.

The overall literature screening and study selection process used in this review is presented in Figure 1.

**Figure 2:**
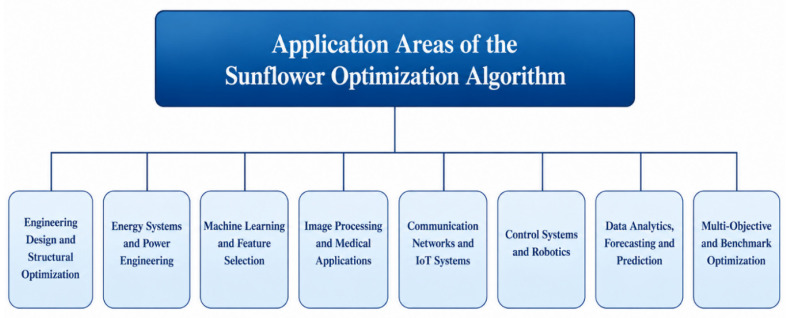
Application areas of the SFO.

The included studies were classified into eight main categories according to their application areas and research focuses. This classification was created to systematically evaluate the usage trends and research intensity of SFO in different disciplines. The application taxonomy developed in this study is presented in Figure 2.

It should be noted that some degree of conceptual overlap naturally exists among application domains, particularly in interdisciplinary fields such as machine learning, image processing, forecasting, and feature selection. To ensure consistency, each study was assigned to a single category according to its primary application objective and dominant research focus rather than the individual techniques employed. For example, studies primarily addressing forecasting problems were classified under Data Analytics, Forecasting and Prediction, whereas studies focusing on feature selection or machine learning model development were categorized under Machine Learning and Feature Selection, even when similar methodological approaches were employed.

The studies under each category were comparatively evaluated and systematically presented in tables. In this context, the studies were classified according to reference information (Ref.), application domain (Domain), specific application area (Application), and the type of SFO used [standard, hybrid, or modified]. In this way, the study analyzed in which problem types SFO was more commonly applied across different disciplines and which algorithm types were more frequently preferred. In addition, this classification helped reveal current research trends, application diversity, and potential future research directions in the literature.

To ensure a consistent classification framework, the reviewed studies were categorized into standard, modified, and hybrid SFO groups according to the algorithmic structure employed. Studies using the original SFO algorithm without any structural modification or integration with other methods were classified as standard SFO. Studies introducing new search mechanisms, update strategies, parameter adaptation techniques, or other algorithmic improvements while preserving the main SFO framework were classified as modified SFO. Studies integrating SFO with other optimization algorithms, machine learning models, deep learning frameworks, feature selection methods, or complementary computational techniques were classified as hybrid SFO. When a study simultaneously contained both modification and integration components, the hybrid classification was prioritized.

Through this methodological framework, the development of SFO in different engineering and AI fields was systematically evaluated, and a comprehensive evaluation framework was established regarding the strengths, limitations, and future research potential of the algorithm.

## 4. Application Areas of SFO

### 4.1. Engineering Design and Structural Optimization

SFO algorithm is a nature-inspired metaheuristic method that has gained increasing attention in the recent literature for solving engineering design and structural optimization problems. The design process of engineering systems involves complex optimization tasks that require the simultaneous consideration of multiple criteria such as performance, strength, efficiency, and cost. Therefore, finite element modeling, experimental analyses, and multi-objective optimization approaches are widely employed in the design and analysis of engineering systems. Within this framework, SFO has been applied to a broad range of engineering problems. The literature indicates that SFO has been utilized in various applications, including structural damage identification and SHM, composite structure design optimization, performance enhancement of mechanical systems and engineering components, and optimization of manufacturing processes and machining parameters. Moreover, SFO has also been employed in material and construction engineering, industrial system optimization, and electronic circuit design. In this section, the applications of SFO in engineering design and structural optimization are reviewed and systematically classified based on different problem types reported in the literature.

Structural damage identification and SHM are important for ensuring the safety and durability of engineering structures. The literature shows a shift from classical methods toward modeling and optimization-based approaches. Within this framework, nature-inspired algorithms, especially SFO algorithm, have been widely used for damage detection problems. Studies report that SFO has been applied in truss structures using multi-objective optimization based on natural frequencies and mode shapes [23], and in composite plates by combining modal shape curvature methods with SFO to identify both damage location and severity [24]. Moreover, SFO-based approaches have shown effective performance in model updating studies that combine experimental data and finite element models [25]. It has also been reported that hybrid models integrating SFO with ANN and RSM can improve accuracy in crack detection [26]. In addition, different parameter tuning strategies have been proposed to enhance the performance of SFO [27], and earlier studies confirm its effectiveness in multimodal optimization problems for composite laminated structures [12]. Furthermore, comparative studies have shown that SFO provides competitive performance against other optimization algorithms in structural damage identification problems [28].

The optimization of composite materials and advanced engineering structures is an important research area that aims to improve structural performance through the proper selection of design parameters, and in this process, SFO algorithm has been used as an effective optimization tool. For example, in a study aimed at improving the performance of CFRP/GFRP hybrid composite tubes, drop-off locations were determined using design of experiment (DOE) and finite element analyses, and optimal design configurations were obtained using the SFO algorithm [29]. Similarly, in a study conducted on isogrid structures, the SFO algorithm was applied to optimize multiple criteria such as mass, natural frequency, and structural strength simultaneously [30].

The optimization of mechanical systems and engineering components aims to improve system performance through the proper selection of design parameters, and in this process, SFO algorithm has been used as an effective optimization tool. For example, in airless tire design, parametric finite element models and multi-objective optimization approaches were applied, and the SFO algorithm was used to improve criteria such as displacement, stress, and mass [31]. Similarly, in the design of six-degree-of-freedom quasi-zero-stiffness vibration isolators, the SFO algorithm was used to optimize system parameters and achieve low natural frequency values [32]. In addition, in the design of Formula SAE vehicle chassis, the SFO algorithm was evaluated comparatively with other optimization methods within a multi-objective optimization framework supported by AI-based metamodeling [33]. Furthermore, in studies on functionally graded plates, the SFO algorithm was applied together with other methods and showed effective performance in determining optimal design variables [34]. Moreover, the SFO algorithm has also been used in the optimization of origami-based engineering structures [35] and in improving the performance of industrial mechanical equipment [36], demonstrating its applicability across different mechanical systems.

Manufacturing processes and machining parameters are optimized to improve production quality and reduce costs. Performance criteria such as surface roughness, material removal rate, and dimensional accuracy are often optimized together. In this context, DOE, statistical modeling, and optimization methods are widely used, and nature-inspired algorithms such as SFO are applied. Studies show that in nano-second pulsed laser machining of Inconel 718 and metal matrix polycrystalline diamond, parameters such as laser fluence and scanning speed were analyzed, and optimal machining settings were obtained using SFO within a multi-objective framework [37]. SFO has also been applied in electrochemical machining of AISI 4140 steel, where Taguchi and WASPAS methods were combined to optimize surface roughness and material removal rate [38]. Similarly, in the turning process of Ti-6Al-4V ELI alloy, SFO was used with experimental models to optimize tool wear, surface roughness, and material removal rate [39]. In addition, SFO has been used to improve shrinkage and collapsibility properties of resin-bonded sand cores based on Taguchi results [40]. It has also been reported that in FDM-based additive manufacturing, SFO provides effective results in optimizing parameters such as raster orientation, layer thickness, and air gap for better dimensional accuracy [41].

Material and construction engineering studies aim to improve material performance, reduce costs, and support sustainable structures. Applications such as soil improvement and concrete mix design play an important role in practice. In this context, experimental and optimization-based methods are widely used, and algorithms such as SFO are applied to determine optimal material parameters. Studies show that in clay core composites used in embankment dams, Taguchi-based experimental design was used to determine parameters such as maximum dry density and optimum moisture content, and the results were further optimized using SFO to identify the most suitable material composition [42]. Similarly, SFO has been applied in recycled aggregate concrete studies, where it was combined with explainable AI to classify early-age compressive strength and to obtain interpretable rules for mix design [43].

Electronic circuit design and analog filter optimization aim to improve signal processing and communication systems. In analog filter design, circuit parameters strongly affect frequency response, stability, and accuracy. Accordingly, modeling and optimization methods are widely used, and algorithms such as SFO are applied to determine optimal circuit parameters. Studies show that in fractional-order low-pass Butterworth filter design, SFO was used to optimize filter coefficients by minimizing the difference between ideal and estimated responses, leading to high accuracy in analog circuit performance [44].

When studies in engineering design and structural optimization are examined, the literature shows that SFO has been widely used as an effective optimization tool in various engineering problems. In particular, in structural damage identification and SHM, SFO has demonstrated strong performance in inverse problem-based model updating processes. In addition, studies on composite structure design and mechanical system optimization indicate that SFO is effective in solving multi-objective optimization problems. Moreover, SFO has been applied to determine optimal design parameters in different areas such as manufacturing processes, machining optimization, material and construction engineering, and industrial systems. It has also been used in more specific applications, including electronic circuit design. Overall, these findings indicate that SFO provides a robust and versatile optimization approach that can be applied to a wide range of engineering design and structural optimization problems.

The reviewed studies are systematically classified in Table 1 according to their application domains, SFO variants, and publication years in order to present the research trends and application diversity in this field more clearly.

To better illustrate the temporal evolution of research trends in this field, the yearly distribution of standard, hybrid, and modified SFO variants is presented in Table 2.

The yearly distribution of SFO variants in engineering design and structural optimization studies shows that hybrid approaches are the most commonly used methods, with a total of 13 studies. Standard SFO approaches account for 6 studies, while modified variants appear in 4 studies. The studies were published between 2019 and 2025, with higher numbers of publications observed in 2021, 2022, and 2025.

### 4.2. Energy Systems and Power Engineering

SFO algorithm has emerged as a promising metaheuristic approach for addressing complex optimization problems in energy systems and power engineering. Owing to its simple structure and effective exploration–exploitation capability, SFO has been applied across a wide range of applications, including modeling, optimization, and control tasks. Recent studies indicate a clear shift from classical deterministic methods toward metaheuristic-based approaches such as SFO. In this context, SFO has been utilized in diverse domains, including battery systems, renewable energy modeling, hybrid energy systems, smart grids, control applications, and power system optimization. The following sections provide a comprehensive overview of these application areas, highlighting the capabilities, performance characteristics, and practical implications of SFO-based approaches.

Battery modeling and SoC/capacity estimation are important for battery management systems. The literature shows a clear shift from classical model-based and filtering methods to hybrid and adaptive approaches supported by metaheuristic algorithms. In this framework, SFO algorithm is used to improve accuracy and modeling performance in online parameter identification and SoC/capacity estimation [13,45,46,47]. Moreover, SFO-based methods have developed from simple parameter identification to more comprehensive applications such as filter tuning and capacity estimation. Optimizing noise covariance matrices improves the stability of filter-based methods and provides more reliable SoC estimation [14], while low error rates and high accuracy have been achieved in capacity estimation problems [15].

PV systems, fuel cells, and power converter modeling and optimization are important research areas in energy systems. The literature shows a clear shift from classical methods toward approaches supported by metaheuristic algorithms. In this framework, SFO algorithm has been effectively used for accurate parameter estimation of PV models [16,17], for error minimization and parameter identification in fuel cell models [20], and for improving maximum power extraction in MPPT applications as well as enhancing the performance of power converter systems [18,19].

Techno-economic design and optimization of hybrid energy systems such as CCHP and micro-CCHP are important research areas for improving energy efficiency and system performance. The literature shows a clear shift from traditional design approaches toward optimization-based methods supported by metaheuristic algorithms. In this context, SFO algorithm has been effectively used to improve system design and energy efficiency in hybrid energy systems [21,22]. In addition, it has shown successful results in technology selection and multi-objective performance optimization of fuel cell-based systems [48]. Moreover, SFO has also been applied effectively in economic analysis and system sizing problems of hybrid PV–fuel cell systems [49].

Energy management and scheduling in smart grids and microgrids are important research areas for the efficient and reliable operation of energy systems. The literature shows a shift from rule-based methods toward optimization approaches supported by metaheuristic algorithms in energy management problems. In this case, SFO algorithm has been successfully applied to problems such as energy scheduling, demand-side management, and efficient use of energy storage systems [50,51,52,53,54]. Moreover, SFO-based approaches have also shown effective results in more complex energy management scenarios, including electric vehicle integration, incorporation of renewable energy sources, and multi-stage scheduling problems [55,56,57,58].

Controller design, frequency regulation, and stability enhancement are key research areas in modern power systems, especially with the growing integration of renewable energy sources. The literature shows a shift from classical tuning methods toward more flexible and adaptive approaches supported by metaheuristic algorithms. In this regard, SFO has been widely used for tuning PID controllers and their variants; for example, SFO-based methods improve frequency regulation performance using fractional-order and fuzzy controllers [59,60,61]. Moreover, SFO has been applied in microgrid control and autonomous operation to enhance system stability under varying load and fault conditions [62,63]. Comparative studies indicate that SFO provides competitive performance in terms of transient response [64], and it has been effectively used in power system stabilizer (PSS) design to improve oscillation damping [65]. In addition, SFO has been applied to automatic generation control (AGC) problems in deregulated power markets, leading to improved system performance [66].

Distribution network reconfiguration, topology optimization, and distributed generation placement are important topics for reducing power losses and improving system efficiency. The literature shows a clear shift from classical deterministic methods to more flexible and effective optimization approaches supported by metaheuristic algorithms. In this context, SFO algorithm has been successfully used in distribution network reconfiguration problems to reduce power loss and determine the optimal topology [67,68,69]. In addition, SFO-based approaches improve system performance by providing effective solutions for the optimal location and sizing of distributed generation units [70,71,72]. Furthermore, comparative studies evaluate the performance of SFO together with other metaheuristic algorithms [73,74,75], and it has been shown that SFO can produce reliable solutions even under uncertain conditions [76].

OPF, reactive power dispatch, and economic-emission load dispatch are key problems for the safe and efficient operation of power systems. The literature shows a shift from classical optimization methods toward more flexible approaches supported by metaheuristic algorithms. In this regard, SFO has been applied in OPF problems to minimize generation cost and improve overall system performance, and it has been evaluated in comparison with other metaheuristic algorithms [77,78,79]. Moreover, SFO has been used in reactive power dispatch problems to improve voltage profiles and reduce power losses, with its performance compared across different algorithms [80,81]. In addition, hybrid and improved SFO methods have demonstrated enhanced solution quality and improved performance across different problem structures [82,83].

Overall, the reviewed studies indicate that SFO algorithm is a flexible and promising approach for addressing a wide range of problems in energy systems and power engineering. SFO has been applied in various domains, including modeling, optimization, control, and system operation. The literature suggests that SFO can provide competitive performance and satisfactory results in many applications, particularly when compared with classical methods. However, its performance may vary depending on the problem structure, and in some cases, other metaheuristic algorithms may achieve better results. In addition, improved and hybrid versions of SFO have been shown to enhance solution quality, especially in complex and uncertain environments. Overall, SFO offers a practical and adaptable framework with significant potential for future research and real-world applications.

To provide a clearer overview of the current research trends, the reviewed studies are categorized based on their application domains, SFO variants, and publication years, as presented in Table 3.

Table 4 presents the yearly distribution of standard, hybrid, and modified SFO variants in energy systems and power engineering studies.

The yearly distribution of SFO variants in energy systems and power engineering studies shows that standard approaches were used in 22 studies, while hybrid and modified variants were reported in 9 and 18 studies, respectively. The studies were published between 2019 and 2025. The highest number of studies was observed in 2021, followed by 2020, 2022, and 2024.

### 4.3. Machine Learning and Feature Selection

The field of machine learning and feature selection has shown significant progress in recent years, especially with the integration of metaheuristic optimization techniques. In this context, SFO algorithm has emerged as a flexible approach that provides effective results across a wide range of applications, including feature selection, biomedical data analysis, DL optimization, explainable AI, and smart agriculture systems. In the literature, there is a clear shift from traditional methods to metaheuristic and hybrid approaches, and SFO has been shown to improve model accuracy, enhance search efficiency, and in some cases produce more interpretable solutions. However, the performance of SFO may vary depending on the problem structure, and recent studies have focused on improved and hybrid variants to address these limitations. Accordingly, in this section, the related studies are systematically examined under thematic subgroups.

Feature selection algorithms and methodological Improvements is an important research area that aims to reduce irrelevant features in high-dimensional data and improve model performance and computational efficiency. In the literature, there is a clear shift from classical methods to metaheuristic and hybrid approaches, where SFO-based methods have played an effective role in this transformation. In this context, the hybrid structure integrating SFO with RFE has achieved higher accuracy and stability in biomarker discovery [84], while binary SFO variants enhanced with chaotic maps and Lévy flights have been shown to produce more effective searches and higher-quality, lower-dimensional feature subsets [85].

Biomedical and healthcare machine learning applications is an important field where data-driven decision support systems are developed for disease diagnosis and biomedical data analysis. In the literature, there is a shift from classical methods to AI- and metaheuristic-supported models, and SFO has been shown to improve model performance; for example, gradient boosting models optimized with SFO achieved high accuracy in diabetes prediction [86], while SFO-based DL approaches have produced strong results in Parkinson’s disease classification [87].

DL and NLP-based intelligent systems is an important field where DL and NLP techniques are used together. In the literature, there is a shift toward metaheuristic optimization to improve model performance, and SFO has been shown to be effective in hyperparameter optimization, leading to improved emotion recognition performance [88].

Explainable AI and interpretable machine learning is an important research area that aims to make model decisions understandable and transparent. In the literature, there is a shift from black-box models to explainable and rule-based approaches, and SFO has been shown to be effective in building interpretable models; for example, rule-based and explainable results have been obtained using chaotic SFO [89], and SFO-based approaches have also provided effective results in the interpretable analysis of biomedical data [90].

Agriculture and smart environment applications is an important field focused on developing intelligent solutions to improve efficiency and sustainability in agricultural and environmental systems. In the literature, there is a shift from traditional methods to AI- and metaheuristic-supported systems, where SFO has been shown to enhance prediction and optimization performance; for example, SFO-based approaches achieved high accuracy in plant growth prediction [91], and when integrated with DL, they produced effective results in smart irrigation systems [92].

Overall, the reviewed studies demonstrate that SFO algorithm is a flexible and competitive approach for addressing a wide range of machine learning and feature selection problems. In particular, SFO has shown strong capability in enhancing model accuracy, improving search efficiency, and generating compact feature subsets across various application domains. However, its performance is often problem-dependent, and challenges such as premature convergence and limited population diversity may affect its effectiveness in complex search spaces. To address these limitations, recent studies increasingly focus on improved and hybrid SFO variants, incorporating mechanisms such as chaotic maps, Lévy flights, and learning-based strategies. These developments highlight the strong potential of SFO as a promising optimization framework for future research and real-world applications.

For a more structured evaluation of the literature, the reviewed studies are grouped according to their application domains, SFO variants, and publication years, as summarized in Table 5.

Table 6 presents the yearly distribution of standard, hybrid, and modified SFO variants in machine learning and feature selection studies.

The yearly distribution of SFO variants in machine learning and feature selection studies shows that hybrid approaches were used in 6 studies, while modified and standard variants were reported in 2 and 1 studies, respectively. The studies were published between 2024 and 2026, with a balanced distribution across the publication years.

### 4.4. Image Processing and Medical Applications

The field of image processing and medical applications is a broad and dynamic research area that involves analyzing, processing, and applying image data across various domains. In the literature, a clear shift can be observed from traditional image processing techniques to DL and metaheuristic optimization-based hybrid models, where SFO is effectively used, especially for parameter optimization, feature selection, and improving model performance. In this context, the studies presented below are examined under different sub-application areas such as medical image analysis, image compression, reconstruction, and security, in order to systematically reveal the usage trends and contributions of SFO.

Medical image analysis, disease diagnosis, and clinical decision support is an important research area focused on the early detection and classification of diseases. In the literature, a clear shift can be observed from classical methods to DL and metaheuristic optimization-based hybrid models, where SFO is effectively used for feature selection and hyperparameter optimization; for example, hybrid deep transfer learning models for oral cancer diagnosis [93], optimized CNN-based models for brain tumor classification [94,95], feature selection-based approaches for neurodegenerative diseases such as Parkinson’s and Alzheimer’s [96,97], optimized DL models for lung nodule and cancer detection [98,99], a Levy flight-supported SFO-based CNN model for heart disease prediction [100], SFO-optimized machine learning and DL models for mammogram and breast tumor diagnosis [101,102], and biomarker-based liver cancer detection approaches [103] have achieved high accuracy and reliability.

Medical image compression and reconstruction plays a crucial role in improving storage and transmission efficiency while minimizing quality loss in medical images. In the literature, a shift can be observed from traditional compression methods to optimization-based and hybrid intelligent approaches, where SFO is effectively used to enhance compression performance and reconstruction quality; for example, ROI and non-ROI based MRI image compression methods [104] and Taylor-SFO-based compressive sensing approaches for image compression and reconstruction [105] have achieved high efficiency and improved image quality.

Agricultural and plant disease image analysis focuses on the early detection of plant diseases and improving agricultural productivity. In the literature, a shift can be observed from traditional methods to DL and optimization-based hybrid models, where SFO is effectively used, particularly for parameter optimization; for example, a DL model supported by attention mechanisms for pomegranate disease detection [106], optimized SVM-based approaches for paddy leaf disease classification [107,108], DL-based models for early detection of crop issues [109], IoT-based classification of rice leaf diseases [110], detection and classification of groundnut leaf diseases [111], and optimized neural network-based models for tomato leaf disease segmentation [112] have achieved high accuracy and effectiveness.

Remote sensing and geospatial image analysis focuses on analyzing Earth surface features using satellite images. In the literature, a shift can be observed from traditional methods to DL and optimization-based models, where SFO is mainly used for parameter optimization; for example, optimized CNN-based methods for multi-class remote sensing image retrieval [113] and optimized DL models for land cover classification using SAR and Landsat images [114] have achieved high accuracy and improved performance.

Image retrieval, general image classification, and vision-based recognition focus on the classification and analysis of images. In the literature, a shift can be observed from traditional methods to DL and optimization-based models, where SFO is mainly used for parameter optimization; for example, multi-feature extraction and selection strategies for video-based human action recognition [115], optimization-supported approaches in content-based image retrieval systems [116], license plate recognition systems for smart city applications [117], and optimized neural network-based models for general image classification problems [118] have achieved high accuracy along with significant improvements in performance metrics.

Image enhancement, restoration, compression, and multimedia security focus on improving image quality and ensuring secure data transmission. In the literature, a shift can be observed from traditional methods to optimization-based models, where SFO is mainly used for parameter optimization; for example, DL-based approaches for video forgery detection [119], denoising methods for multispectral images [120], compressive sensing-based image compression and reconstruction techniques [121], digital image watermarking methods [122], and chaotic system-based image encryption approaches [123] have achieved high performance and significant improvements in security metrics.

Overall, the reviewed studies demonstrate that SFO algorithm is a flexible and effective approach across various image processing and medical application domains. In particular, SFO is widely used to improve model performance through parameter optimization and feature selection, often in combination with DL and hybrid frameworks. The results show that SFO-based methods generally achieve higher accuracy, better efficiency, and more reliable outcomes compared to traditional approaches. However, the performance of SFO may vary depending on the problem structure and data characteristics. Therefore, recent studies tend to develop improved and hybrid variants of SFO to enhance its search capability and robustness. These findings highlight the strong potential of SFO for future research and practical applications in image-based systems.

To present the diversity of application areas more clearly, the reviewed studies are categorized according to their domains, SFO variants, and publication years, as shown in Table 7.

Table 8 presents the yearly distribution of standard, hybrid, and modified SFO variants in image processing and medical applications studies.

The yearly distribution of SFO variants in image processing and medical applications studies shows that hybrid approaches were used in 18 studies, while modified and standard variants were reported in 10 and 3 studies, respectively. The studies were published between 2020 and 2026. The highest number of studies was observed in 2023, followed by 2022, 2024, and 2025.

### 4.5. Communication Networks and IoT Systems

The communication networks and IoT systems field is a dynamic research area that covers data transmission, security, energy efficiency, and overall system performance. In recent years, a clear shift has been observed from traditional approaches to hybrid models supported by AI and metaheuristic optimization, where SFO algorithm provides effective solutions for key problems such as routing, clustering, intrusion detection, secure communication, and resource allocation. In this context, the related studies are examined across different application areas to present the usage trends of SFO and its contributions to system performance from a systematic perspective.

Energy-efficient clustering, CH selection, and network lifetime optimization are key research areas in WSN/IoT systems, as they directly influence energy consumption and network sustainability. Although the studies in the literature aim at a common objective, they show diversity by focusing on different sub-problems such as CH selection [124,125,126,127,128], clustering and routing integration [129,130,131], and comparative or analytical approaches [132]. In recent years, SFO and its variants have been widely adopted in these problems, and both standalone and hybrid models (e.g., Lévy flight and GWO-based approaches) have been shown to improve the exploration–exploitation balance and reduce the risk of local optima, leading to more stable, energy-efficient, and longer-lasting network structures [124,125,126].

Routing, data collection, data aggregation, and mobile sink optimization are fundamental research areas in WSN/IoT systems, as they directly affect data transmission efficiency and energy consumption. In the literature, these problems are addressed under different subtopics; for example, mobile data collection and path planning approaches [133,134], clustering and routing integration-based methods [135,136], and techniques that jointly consider performance metrics such as data aggregation, reliability, and delay reduction [137,138]. In recent years, SFO algorithm and its hybrid variants have been widely applied to these sub-problems, providing more effective and stable solutions in terms of route optimization, data collection efficiency, and energy balance [133,134,135,136,137,138].

Intrusion detection, attack detection, and trust-aware secure communication focus on ensuring network security and reliable data transmission in IoT/WSN/MANET/IIoT environments. In the literature, a clear shift can be observed from traditional machine learning-based approaches to DL and optimization-based models, where SFO is widely used for feature selection, parameter optimization, and secure routing processes; for example, optimized DL models for advanced threat detection [139], SFO-based intrusion detection and routing approaches in WSN and MANET environments [140,141,142,143,144], trust-aware routing strategies [145,146], and hybrid intelligent intrusion detection models for IIoT systems [147,148] have achieved higher accuracy and improved security performance.

Blockchain- and cryptography-based privacy and security approaches play a critical role in ensuring data integrity, access control, and secure communication in IoT and networked systems. In the literature, a clear shift can be observed from centralized architectures to distributed and smart contract-based systems, where SFO is effectively used for key generation, encryption parameter optimization, and secure data transmission processes; for example, blockchain-based healthcare data security and key management solutions [149,150], encryption and auditing mechanisms in cloud and data storage systems [151,152], and privacy-preserving and secure communication approaches in IoT and VANET environments [153,154] have achieved higher security levels and improved system performance.

Cloud computing, task scheduling, load balancing, and resource allocation are fundamental research areas that directly affect performance and resource efficiency in cloud-based systems. In the literature, a shift can be observed from traditional approaches to optimization-based models, where SFO is effectively applied; for example, dynamic load balancing in cloud environments [155], data transfer and path planning processes [156], optimization-supported model development for cloud-based intrusion detection systems [157], and hybrid metaheuristic-based resource allocation strategies [158] have provided significant improvements in reducing latency, balancing workload, and enhancing model accuracy and overall system performance.

Wireless communication system optimization, particularly in the context of Massive MIMO, antenna selection, spectrum sensing, and SD-IoT, aims to improve communication performance and spectrum efficiency. In the literature, a shift from traditional methods to optimization-based models can be observed, where SFO is effectively utilized; for example, antenna selection optimization in Massive MIMO systems [159,160], spectrum sensing and PAPR reduction processes [161], and controller placement problems in SD-IoT environments [162] have provided significant improvements in channel capacity, energy efficiency, latency reduction, and overall system performance, depending on the problem structure.

IoT-enabled smart agriculture and plant monitoring systems aim to improve agricultural processes and increase productivity through sensor-based data analysis. In the literature, a shift from traditional methods to AI and optimization-based models can be observed, where SFO is effectively used; for example, SFO-based routing and DL models for plant disease prediction and secure data transmission [163,164,165] have achieved high accuracy, improved energy efficiency, and enhanced overall system performance.

VANET and intelligent transportation systems aim to improve vehicle-to-vehicle communication and traffic safety. In the literature, a shift from traditional methods to AI and optimization-based models can be observed, where SFO is effectively used; for example, SFO-supported DL models for driver behavior-based mobility analysis and routing performance evaluation in VANET environments [166] have improved both driver behavior prediction accuracy and overall network communication performance.

Overall, the reviewed studies show that the SFO algorithm is a flexible and effective approach in the field of Communication Networks and IoT Systems. SFO is widely used to improve performance in problems such as routing, clustering, security, and resource management, often in combination with hybrid models. However, its performance may vary depending on the problem structure, and recent studies tend to focus on improved SFO variants. These findings indicate that SFO has strong potential for future applications in this field.

To better present the research distribution and application diversity in this field, the reviewed studies are classified according to their problem domains, SFO variants, and publication years, as presented in Table 9.

Table 10 presents the yearly distribution of standard, hybrid, and modified SFO variants in communication networks and IoT systems studies.

The yearly distribution of SFO variants in communication networks and IoT systems studies shows that hybrid approaches were used in 24 studies, while modified and standard variants were reported in 13 and 6 studies, respectively. The studies were published between 2020 and 2026. The highest number of studies was observed in 2023, followed by 2021 and 2024.

### 4.6. Control Systems and Robotics

The field of control systems and robotics is an important research area that focuses on controlling complex and dynamic systems, improving decision-making processes, and developing autonomous structures. Studies in this field cover a wide range of applications, from radar and signal processing systems to electric vehicles, power systems, and robotic applications. In the literature, there is an increasing shift toward AI and metaheuristic optimization techniques to overcome the limitations of traditional control methods. In this context, approaches such as SFO algorithm are considered promising optimization methods that improve system performance and provide more stable solutions across different application domains.

Radar, signal processing, and estimation systems are important research areas that aim to improve target detection and parameter estimation accuracy under challenging conditions. In the literature, there is a growing shift from traditional methods to metaheuristic approaches such as SFO, especially in the presence of impulsive noise; for example, SFO-based methods developed for simultaneous estimation of target number and angle–range parameters in FDA-MIMO radar systems [167] provide more accurate identification of peaks in the two-dimensional spatial spectrum, leading to significant improvements in system performance.

Electric vehicle and motor control systems are important research areas that aim to improve system stability, energy efficiency, and overall performance. In the literature, a shift from traditional methods to metaheuristic approaches such as SFO algorithm can be observed; for example, in electric vehicle speed control [168], speed–current control and torque ripple reduction in SRM motors [169], and parameter estimation of multi-phase induction motors [170], SFO-based methods improve control performance, reduce error rates, and provide more stable results.

Power systems and microgrid control focus on maintaining system stability and dynamic response under increasing uncertainties due to the integration of renewable energy sources. In recent years, a shift beyond classical control approaches toward AI and metaheuristic-based methods has been observed; in this context, SFO algorithm has emerged as an effective tool in studies such as frequency regulation [171], microgrid PI controller optimization [172], and the evaluation of adaptive control strategies [173], providing lower error and improved system stability.

Robotic motion planning and navigation focus on enabling robots to achieve accurate orientation and safe movement in complex and dynamic environments. In recent years, optimization-based approaches integrated with sensor data have gained importance over classical methods; in this context SFO algorithm has been highlighted as an effective solution for orientation control in humanoid robots [174] and for path planning and obstacle avoidance in wheeled robots [175], supporting more efficient decision-making and improved navigation performance.

Multi-robot systems and advanced robotic optimization focus on enabling coordinated operation and task sharing among robots. In the literature, a shift from classical methods to hybrid AI and metaheuristic-based models can be observed; in this context, SFO algorithm-based approaches provide more flexible, adaptive, and high-performance solutions for trajectory planning, obstacle avoidance, and decision-making in multi-humanoid robots [176].

Taken together, the reviewed studies indicate that SFO algorithm is a flexible and widely applicable approach in control systems and robotics. It has been used to improve system performance, enhance stability, and address complex and dynamic problems across domains such as radar systems, motor control, power systems, and robotics. The findings suggest that SFO-based methods often provide higher accuracy and more reliable outcomes compared to traditional approaches. However, performance may vary depending on the problem structure and system conditions, and issues such as parameter tuning and convergence behavior can still affect the results. Therefore, recent studies increasingly focus on hybrid and improved variants of SFO, highlighting its strong potential for future research and real-world applications.

To summarize the main research directions in this field, the reviewed studies are categorized according to their application domains, SFO variants, and publication years, as presented in Table 11.

Table 12 presents the yearly distribution of standard, hybrid, and modified SFO variants in control systems and robotics studies.

The yearly distribution of SFO variants in control systems and robotics studies shows that standard approaches were used in 5 studies, while hybrid and modified variants were reported in 3 and 2 studies, respectively. The studies were published in 2021, 2022, 2024, and 2025. The highest number of studies was observed in 2021, followed by 2022.

### 4.7. Data Analytics, Forecasting and Prediction

The data analytics, forecasting and prediction domain is an important research area that focuses on analyzing complex data and making accurate future predictions. In the literature, there is a clear shift from traditional methods to hybrid models based on AI and metaheuristic optimization; in this context, SFO algorithm is widely used in different application areas to improve model performance and obtain more reliable results. In this section, the usage trends and contributions of the SFO algorithm in various data analytics and prediction-oriented subdomains are systematically examined.

The environmental, hydrological and climate forecasting domain focuses on water resource management, modeling of climate variables, and accurate prediction of environmental processes. In the literature, there is a clear shift from traditional statistical methods to hybrid models based on AI and metaheuristic optimization; in this context, SFO-based approaches are widely used in different applications such as streamflow prediction [177,178], evaporation modeling [179], soil temperature prediction with uncertainty analysis [180], and lake water level forecasting [181], generally improving model accuracy and reducing uncertainty.

The energy systems, industrial process and materials prediction domain is an important research area that focuses on the accurate and efficient modeling of energy production and consumption processes, industrial operations, and material properties. In this context, SFO-based methods have been applied in various applications such as CO/CO_2_ prediction in gasification processes [182], electrochemical property prediction of materials [183], electricity demand forecasting [184], and modeling of industrial production processes [185], generally improving model accuracy and providing more reliable predictions.

The disaster, hazard and risk prediction domain is a critical research area that aims to reduce human and economic losses by predicting natural disasters and risky events in advance. In the literature, there is a shift from traditional statistical approaches to hybrid models based on AI and metaheuristic optimization; in this context, SFO-based methods have been applied in seismic risk prediction in mining activities [186] and in multi-disaster scenarios such as flood and earthquake detection [187], improving model performance and enhancing the effectiveness of early warning systems.

The civil, geotechnical and transportation prediction domain is an important research area that focuses on the accurate modeling and prediction of infrastructure systems, soil behavior, and traffic flow. In this context, SFO-based methods have been applied in applications such as pile settlement prediction [188] and traffic flow prediction at signalized intersections [189], improving model accuracy and providing more effective prediction performance.

The financial and business intelligence prediction domain is an important research area that focuses on analyzing financial risks, customer behavior, and business processes to develop effective decision support systems. In this context, SFO-based methods have been applied in applications such as financial crisis prediction [190] and customer churn prediction [191], improving model performance and providing more reliable predictions.

The text analytics, summarization and information retrieval domain is an important research area that focuses on processing large-scale text data, extracting meaningful information, and summarizing multiple documents. In this context, SFO-based methods have been applied in applications such as multi-document summarization [192] and cross-lingual information retrieval with query optimization [193], improving model performance and providing more effective results.

The sentiment analysis and social media analytics domain is an important research area that focuses on analyzing user opinions, emotions, and trends from social media data. In this context, SFO-based methods have been applied in applications such as sentiment analysis on social media data [194] and tweet-based sentiment classification for global events like COVID-19 [195], improving model accuracy and providing more effective analysis results.

The medical data analytics and clustering domain is an important research area that focuses on analyzing large and complex medical data, extracting meaningful patterns, and developing decision support systems. In this context, SFO-based methods have been applied in medical data clustering processes [196,197], improving model performance by identifying more accurate cluster centers and enhancing analysis results.

The marketing, advertising and user-oriented predictive analytics domain is an important research area that focuses on analyzing user behavior, providing personalized services, and developing targeted advertising strategies. In this context, SFO-based methods have been applied in location-based advertising and user-oriented prediction systems [198], improving model performance and providing more effective results.

The computational intelligence infrastructure and scheduling analytics domain is an important research area that focuses on resource management, task scheduling, and performance optimization in large-scale computing systems. In this context, SFO-based methods have been applied in cloud computing environments for task scheduling and improving system efficiency [199], enhancing performance and enabling more efficient resource utilization.

The predictive classification in applied data mining domain is an important research area that focuses on extracting meaningful patterns from different datasets and developing accurate classification models. In this context, SFO-based methods have been applied in applied data mining problems [200], improving model performance and providing more reliable results.

Taken together, the reviewed studies demonstrate that SFO algorithm is a versatile and widely applicable approach across a broad range of data analytics, forecasting, and prediction applications. SFO is commonly used to improve model accuracy, optimize parameters, and enhance the performance of hybrid machine learning and DL models across different domains. The findings indicate that SFO-based methods often achieve improved predictive performance and more stable results. However, the performance of SFO may vary depending on the problem structure and data characteristics, and challenges such as sensitivity to parameter settings and convergence behavior may still affect the results. Therefore, recent studies increasingly focus on improved and hybrid versions of SFO, highlighting its strong potential for future research and real-world applications.

To provide a comprehensive overview of the research trends in this field, the reviewed studies are categorized according to their application areas, SFO variants, and publication years, as summarized in Table 13.

Table 14 presents the yearly distribution of standard, hybrid, and modified SFO variants in control systems and robotics studies.

The yearly distribution of SFO variants in data analytics, forecasting and prediction studies shows that hybrid approaches were used in 21 studies, while modified variants were reported in 3 studies. No standard SFO approach was identified in the reviewed studies. The studies were published between 2021 and 2025. The highest number of studies was observed in 2021 and 2022, followed by 2023.

### 4.8. Multi-Objective and Benchmark Optimization

The Multi-Objective and Benchmark Optimization field focuses on improving the performance of optimization algorithms and providing more effective solutions for different types of problems. In this context, the literature shows that studies are mainly addressed within hybrid and improved algorithm design, multi-objective optimization approaches, and application-based performance evaluation; the following section presents studies on the use of the SFO algorithm within these three main approaches.

The hybrid and improved metaheuristic algorithm design approach focuses on developing hybrid and enhanced structures to improve the performance of metaheuristic algorithms. In this context, hybrid approaches in which the SFO algorithm is combined with different methods [201] aim to enhance search capability and solution quality; providing faster convergence and higher accuracy, and thus demonstrating the effectiveness of SFO-based hybrid structures.

The multi-objective optimization and pareto-based approaches focus on developing Pareto-based solutions to optimize multiple objective functions simultaneously. In this context, multi-objective versions of the SFO algorithm [202] aim to improve solution diversity and convergence performance; enhancing the quality of the Pareto front and demonstrating the effectiveness of SFO-based multi-objective optimization approaches.

The benchmark-based validation in applied optimization frameworks approach focuses on evaluating optimization algorithms within real-world applications and validating their performance using standard benchmark functions. In this context, studies where the SFO algorithm is used in application-based models [203] aim to improve model performance; supported by experimental results, they demonstrate the effectiveness of SFO-based optimization approaches across different application domains.

Taken together, the reviewed studies show that the SFO algorithm is a flexible and effective approach for multi-objective and optimization-oriented problem settings. It has been applied in hybrid structures, multi-objective models, and application-based systems to improve search capability, solution quality, and overall performance. The results generally indicate that SFO-based methods provide improved convergence behavior, enhanced solution quality, and more reliable outcomes compared to traditional approaches. However, their performance may depend on the problem structure and parameter settings. Therefore, recent studies increasingly focus on hybrid and improved versions of SFO, highlighting its strong potential for future research and real-world applications.

To present the main optimization-oriented research directions more clearly, the reviewed studies are grouped according to their optimization frameworks, SFO variants, and publication years, as summarized in Table 15.

Table 16 presents the yearly distribution of standard, hybrid, and modified SFO variants in multi-objective and benchmark optimization studies.

The yearly distribution of SFO variants in multi-objective and benchmark optimization studies shows that hybrid approaches were used in 2 studies, while modified variants were reported in 1 study. No standard SFO approach was identified in the reviewed studies. The studies were published in 2021, 2023, and 2025.

## 5. Variants and Hybrid Approaches

### 5.1. Distribution of Standard, Hybrid, and Modified SFO Variants

**Figure 3:**
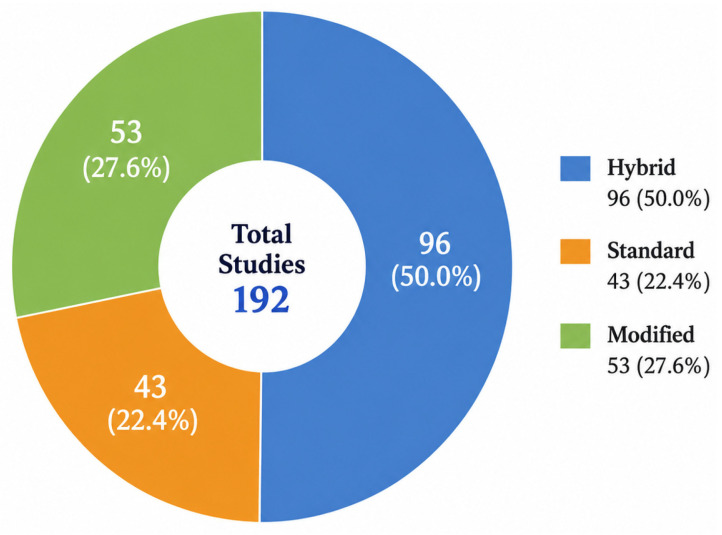
Overall distribution of standard, hybrid, and modified SFO variants.

The reviewed studies demonstrate that SFO algorithm has evolved from a standard optimization approach into various hybrid and modified structures across different application domains. Based on the reviewed literature, hybrid SFO approaches represent the most commonly used category, followed by modified and standard variants. This distribution indicates that researchers increasingly tend to improve the exploration capability, convergence behavior, and solution quality of the standard SFO algorithm through hybridization and modification strategies. To provide a clearer overview of the general research tendency, the overall distribution of standard, hybrid, and modified SFO variants is presented in Figure 3.

The literature also shows that the distribution of SFO variants differs depending on the application domain. In engineering design and structural optimization studies, hybrid approaches were used in 13 studies, while standard and modified variants were reported in 6 and 4 studies, respectively [12,23,24,25,26,27,28,29,30,31,32,33,34,35,36,37,38,39,40,41,42,43,44]. Similarly, in communication networks and IoT systems, hybrid approaches were dominant with 24 studies, followed by modified variants with 13 studies and standard approaches with 6 studies [124,125,126,127,128,129,130,131,132,133,134,135,136,137,138,139,140,141,142,143,144,145,146,147,148,149,150,151,152,153,154,155,156,157,158,159,160,161,162,163,164,165,166]. In image processing and medical applications, hybrid SFO structures were also widely preferred, accounting for 18 studies, while modified and standard approaches were used in 10 and 3 studies, respectively [93,94,95,96,97,98,99,100,101,102,103,104,105,106,107,108,109,110,111,112,113,114,115,116,117,118,119,120,121,122,123].

In contrast, some domains showed a relatively balanced distribution between standard and modified approaches. For example, in energy systems and power engineering studies, standard SFO approaches were reported in 22 studies, while modified and hybrid variants appeared in 18 and 9 studies, respectively [13,14,15,16,17,18,19,20,21,22,45,46,47,48,49,50,51,52,53,54,55,56,57,58,59,60,61,62,63,64,65,66,67,68,69,70,71,72,73,74,75,76,77,78,79,80,81,82,83]. This distribution indicates that the original structure of SFO is still actively preferred in energy-related optimization problems.

Moreover, the reviewed studies indicate that hybrid approaches are particularly dominant in machine learning, forecasting, and prediction-oriented applications. In machine learning and feature selection studies, hybrid variants accounted for 6 out of 9 studies [84,85,86,87,88,89,90,91,92]. Similarly, in data analytics, forecasting, and prediction studies, hybrid approaches were used in 21 studies, while no standard SFO approach was identified [177,178,179,180,181,182,183,184,185,186,187,188,189,190,191,192,193,194,195,196,197,198,199,200]. These findings suggest that hybrid structures are more frequently preferred in data-driven and AI-supported applications. Overall, the literature demonstrates a clear research trend toward hybrid and modified SFO structures. This trend indicates that researchers aim to improve the performance of the standard SFO algorithm and adapt it to more complex optimization problems across different engineering and AI domains.

### 5.2. Research Domain Analysis of SFO

**Figure 4:**
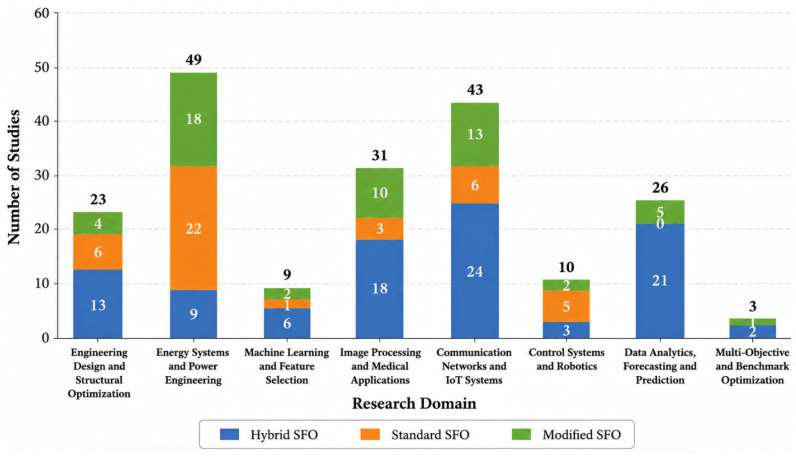
Visual representation of SFO across research domains.

The reviewed studies indicate that the usage trends of SFO variants vary significantly depending on the research domain and application characteristics. In some domains, standard SFO approaches remain dominant, while in others, hybrid and modified structures are more commonly preferred. To provide a clearer overview of these domain-based trends, the distribution of standard, hybrid, and modified SFO variants across different research domains is presented in Figure 4.

Engineering design and structural optimization studies show a strong preference for hybrid SFO approaches [23,24,26,30,31,33,35,36,37,38,40,41,42]. In this domain, hybrid structures were particularly applied in structural damage identification, composite structural optimization, and manufacturing process optimization problems. Standard approaches were generally used in relatively direct optimization problems such as composite plate damage detection and mechanical structure optimization [12,25,28,29,32,34], while modified variants were mainly introduced to improve tuning strategies and optimization performance [27,39,43,44].

In energy systems and power engineering, the literature presents a relatively balanced distribution between standard and modified SFO variants [13,14,15,16,17,18,19,20,21,22,45,46,47,48,49,50,51,52,53,54,55,56,57,58,59,60,61,62,63,64,65,66,67,68,69,70,71,72,73,74,75,76,77,78,79,80,81,82,83]. Standard SFO approaches were widely used in controller design, OPF, distributed generation placement, and battery parameter estimation problems [13,15,18,46,47,48,60,61,63,64,66,67,68,71,73,74,75,77,78,79,80,81]. Modified approaches were particularly preferred in scheduling, network optimization, and economic dispatch problems [16,20,22,45,49,50,51,54,55,56,57,59,65,69,70,72,76,83]. Hybrid structures were generally applied in MPPT systems, energy scheduling, and hybrid energy system optimization studies [14,17,19,21,52,53,58,62,82].

Machine learning and feature selection studies demonstrate a clear dominance of hybrid SFO structures [84,85,88,89,90,91]. Hybrid approaches were mainly integrated with machine learning and DL frameworks to improve classification accuracy and feature selection performance. Modified variants were generally used in binary feature selection and explainable AI-based systems [86,92], while standard SFO appeared only in interpretable biological data analysis [87].

A similar trend was observed in image processing and medical applications. Hybrid SFO structures were widely integrated with CNN, DL, transfer learning, and image processing frameworks [93,95,97,98,100,103,104,106,110,111,114,115,117,118,119,121,122,123]. Modified variants were mainly preferred in optimization-oriented segmentation, compression, and disease classification problems [94,99,101,102,105,107,108,112,113,116], while standard approaches appeared in a limited number of studies [96,109,120].

Communication networks and IoT systems also demonstrate a strong trend toward hybrid approaches [126,127,129,133,134,135,136,138,139,144,145,146,150,152,153,155,158,159,160,161,163,164,165,166]. Hybrid SFO structures were particularly used in routing optimization, clustering, intrusion detection, and wireless communication problems. Modified variants were mainly employed in secure communication, routing optimization, and blockchain-supported systems [124,125,128,130,131,132,137,142,149,151,156,157,162], while standard approaches appeared less frequently [140,141,143,147,148,154].

In control systems and robotics, standard SFO approaches remained relatively dominant [168,170,172,174,175]. Hybrid approaches were mainly applied in motor control and robotic optimization problems [169,173,176], while modified variants were used in radar systems and frequency regulation applications [167,171].

In forecasting and prediction studies, hybrid approaches clearly dominated the literature [177,179,180,181,182,183,184,185,186,187,188,190,191,192,193,194,195,196,197,198,200]. These hybrid structures were integrated with machine learning, DL, ANFIS, LSTM, GAN, and RNN models. Modified variants appeared only in a limited number of prediction problems such as streamflow prediction, traffic flow prediction, and task scheduling [178,189,199]. No standard SFO approach was identified in this category.

Multi-objective and benchmark optimization studies also demonstrate a tendency toward hybrid and modified SFO structures. Hybrid approaches were mainly used in hybrid metaheuristic algorithm development and application-based optimization frameworks [201,203], while modified structures were introduced in constrained multi-objective optimization and Pareto-based optimization problems [202]. No standard SFO approach was identified in this category.

Table 17 presents a numerical summary of the distribution of standard, hybrid, and modified SFO variants across different research domains.

Overall, the reviewed studies indicate that hybrid SFO structures are particularly dominant in AI, communication, and prediction-oriented applications, while standard approaches remain more common in control systems and several energy optimization problems. This distribution highlights the increasing tendency toward hybridization in complex and data-driven optimization tasks.

### 5.3. Hybrid SFO Structures and Integration Trends

The reviewed studies indicate that hybrid SFO structures are widely developed by integrating the SFO algorithm with machine learning, DL, optimization, and statistical modeling techniques. These integrations were mainly introduced to improve optimization performance, classification accuracy, convergence behavior, and solution quality across different application domains.

In engineering applications, hybrid SFO approaches were combined with ANN, RSM, and finite element analysis frameworks. For example, ANN- and RSM-supported hybrid SFO models were used in crack identification problems in laminated composite structures [26]. Similarly, hybrid SFO structures were applied together with multi-objective optimization frameworks in structural and mechanical design problems [30,31,33].

In energy systems and power engineering, hybrid SFO approaches were integrated with EKF, MPPT systems, and hybrid energy management structures. SFO-optimized EKF approaches were applied for battery state-of-charge estimation [14], while hybrid SFO-based methods were used in MPPT and reactive power control applications [19]. Hybrid structures also appeared in energy scheduling and hybrid power system optimization studies [21,52,53,58,62,82].

Machine learning and feature selection studies demonstrated a strong integration trend between SFO and AI models. Hybrid SFO structures were combined with RFE for transcriptomic biomarker selection [84], chaotic and Lévy-flight binary mechanisms for feature selection [85], attention-based DL systems for emotion recognition [88], XAI-based frameworks for food drying performance analysis [89], explainable analysis of bovine uterine gas data [90], and crossover-boosted prediction models for seed germination forecasting [91].

A similar trend was observed in image processing and medical applications. Hybrid SFO structures were integrated with deep transfer learning [93], CNN models [95,97,100], deep belief networks [103], and various DL frameworks for disease diagnosis and image analysis [106,110,111,114,115,118,119,121,122,123]. These studies generally aimed to improve classification accuracy, feature extraction capability, and overall model performance.

Communication networks and IoT systems also showed extensive use of hybrid SFO structures. In this domain, SFO was combined with GWO [126], DE [138], DL models [139,148], autoencoders [145], and blockchain-based systems [150,153]. Hybrid structures were mainly developed to improve routing efficiency, intrusion detection performance, clustering quality, and communication security.

Forecasting and prediction studies revealed one of the strongest hybridization trends in the reviewed literature. Hybrid SFO structures were integrated with MLP [177,179,188], ANFIS [181], LSTM [185], GAN and RNN architectures [192], BiLSTM [194], and DL frameworks for disaster prediction and sentiment analysis [187,191,195]. These integrations generally focused on improving prediction accuracy and model stability.

Overall, the reviewed studies demonstrate that hybrid SFO structures are increasingly integrated with AI, DL, optimization, and statistical modeling approaches. This trend indicates that hybridization has become one of the major research directions in the development of SFO-based optimization frameworks.

### 5.4. Modified SFO Structures and Improvement Strategies

The reviewed literature indicates that modified SFO approaches were mainly developed to improve search capability, convergence performance, optimization accuracy, and population diversity. Different modification strategies were proposed depending on the application domain and problem structure.

In engineering design and structural optimization, modified SFO approaches focused on improving tuning mechanisms and optimization performance [27,39,43,44]. In particular, improved tuning strategies were proposed for structural damage identification problems [27], while modified approaches were also used in machining optimization and analog filter design applications [40,44].

In machine learning and feature selection studies, modified SFO structures were mainly used in explainable artificial intelligence and deep learning-based applications. The modified SFO approach was employed for diabetes diagnosis together with SHAP-supported machine learning models [86] and was also applied to the optimization of deep CNN architectures for smart irrigation control systems [92].

In image processing and medical applications, modified SFO approaches were introduced for segmentation, compression, disease diagnosis, and optimization-oriented image analysis tasks [94,99,101,102,105,107,108,112,113,116]. In some studies, modified structures were combined with Lévy-flight mechanisms [100] or chaotic systems [123] to improve optimization performance and robustness.

In communication networks and IoT systems, modified SFO approaches were mainly used in routing optimization, secure communication, blockchain systems, and clustering problems [124,125,128,130,131,132,137,142,149,151,156,157,162]. Improved SFO structures were proposed for CH selection [125], network lifetime maximization [124], and secure communication frameworks [149,151].

Energy systems and power engineering studies also included several modified SFO approaches for scheduling, dispatch, and optimization problems [16,20,22,45,49,50,51,54,55,56,57,59,65,69,70,72,76,83]. These approaches mainly focused on improving optimization accuracy and solution quality in complex energy management problems.

Moreover, multi-objective optimization studies introduced specialized modified SFO structures such as MOSFO for constrained multi-objective optimization and Pareto front generation [202]. This indicates that modified SFO approaches are also increasingly developed for advanced optimization frameworks.

Overall, the reviewed studies demonstrate that modified SFO structures mainly focus on improving convergence behavior, optimization accuracy, search diversity, and solution stability across different application domains.

### 5.5. General Evaluation of SFO Evolution Across Domains

**Figure 5:**
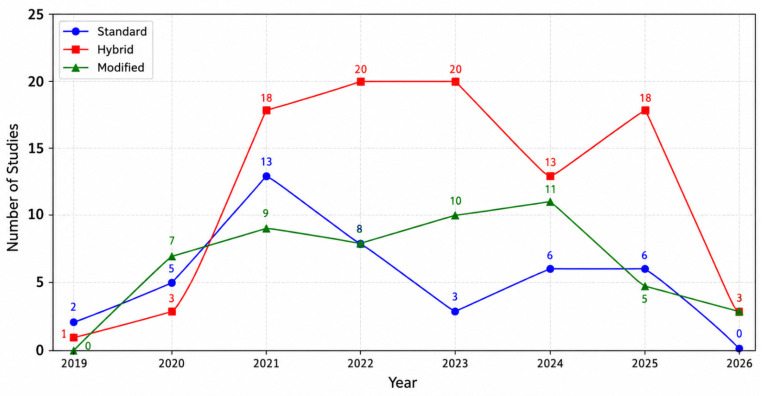
Temporal evolution of standard, hybrid, and modified SFO variants.

The reviewed literature demonstrates that the SFO algorithm has evolved considerably across different application domains. While early studies mainly focused on standard SFO approaches in relatively direct optimization problems [12,46,48,66,77], recent studies increasingly introduced hybrid and modified variants to improve optimization performance and adapt the algorithm to more complex systems. To better illustrate the temporal evolution of SFO variants, the yearly distribution of standard, hybrid, and modified approaches is presented in Figure 5.

The literature also indicates that the evolution of SFO differs depending on the application domain. In control systems and several energy optimization problems, standard SFO approaches are still actively used [60,61,63,64,66,68,71,73,74,75,76,77,78,79,80,81,168,170,172,174,175]. In contrast, AI-oriented domains such as machine learning, image processing, IoT systems, and forecasting applications show a strong tendency toward hybrid structures [84,93,95,97,100,106,110,114,115,118,119,126,127,129,133,134,135,136,139,144,145,146,150,153,159,160,161,177].

In addition, the reviewed studies demonstrate that modified SFO approaches are increasingly introduced to improve search capability and convergence performance, especially in optimization-intensive problems [16,22,27,45,54,55,56,57,69,72,83,85,89,99,101,102,105,113,124,125,149,151,156,162,202].

Overall, the literature indicates a clear research trend from standard SFO structures toward hybrid and modified optimization frameworks. This evolution highlights the growing effort to improve the applicability and performance of SFO in increasingly complex engineering, AI, and optimization problems.

## 6. Discussion: Strengths, Limitations, and Practical Considerations

**Figure 6:**
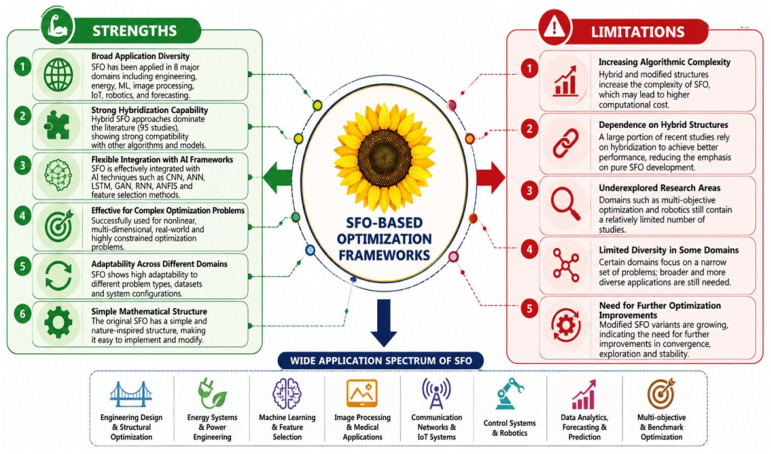
Major strengths and limitations of SFO-based optimization frameworks.

The reviewed studies demonstrate that SFO algorithm has become an increasingly flexible and adaptable optimization method across different engineering and AI domains. The results presented in Section 4 and Section 5 indicate that SFO has evolved from a relatively standard metaheuristic approach into more advanced hybrid and modified optimization structures. In particular, the increasing number of hybrid studies in recent years shows that researchers aim to improve the exploration capability, convergence performance, and solution quality of the algorithm for more complex optimization problems. The major strengths and limitations identified in the reviewed SFO literature are summarized in Figure 6.

One of the major strengths of SFO is its applicability to a wide variety of optimization problems. The reviewed studies show that SFO has been successfully applied in structural optimization, energy management, image processing, communication systems, robotics, forecasting, and machine learning applications. This broad application diversity indicates that the algorithm can effectively handle nonlinear, multi-dimensional, and complex optimization problems. In addition, the relatively simple mathematical structure of SFO provides flexibility for integration with different optimization and AI frameworks.

A further observation from the reviewed literature is that SFO has frequently demonstrated competitive performance when compared with classical population-based metaheuristics such as PSO and GA. Across feature selection, machine learning, energy management, wireless sensor networks, forecasting, and engineering design applications, SFO has often been adopted for problems requiring a balanced exploration–exploitation mechanism in complex nonlinear search spaces. Reported studies commonly indicate improvements in solution quality, convergence behavior, energy efficiency, prediction accuracy, or optimization robustness relative to traditional benchmark algorithms. Furthermore, PSO and GA were identified as the most frequently used baseline methods in the reviewed literature, followed by GWO, WOA, SSA, and TLBO, suggesting that the effectiveness of SFO has been extensively validated against well-established optimization algorithms across diverse application domains. This behavior is largely attributed to the sunflower-inspired search mechanism, which helps maintain population diversity and balance exploration and exploitation, thereby reducing the risk of premature convergence that is commonly reported in classical population-based optimization algorithms.

Another important strength observed in the literature is the strong compatibility of SFO with hybrid optimization frameworks. The reviewed studies clearly show that hybrid SFO approaches became dominant especially in machine learning, image processing, IoT systems, and prediction-oriented applications. This trend suggests that SFO can be effectively integrated with DL models, neural networks, feature selection techniques, and other metaheuristic algorithms. Particularly in data-driven applications, hybrid structures appear to improve classification accuracy, prediction performance, optimization quality, and model stability.

An additional observation emerging from the reviewed machine learning and deep learning studies concerns the architectural role of SFO within AI frameworks. The literature indicates that SFO is most commonly employed as an auxiliary optimization component rather than a complete replacement for conventional learning mechanisms. In the majority of CNN-, LSTM-, BiLSTM-, autoencoder-, and fuzzy system-based applications, SFO is used for hyperparameter optimization, feature selection, parameter estimation, architecture configuration, or model calibration. Gradient-based learning procedures such as backpropagation generally remain responsible for updating network weights, while SFO assists the learning process by identifying improved parameter configurations. Only a limited number of studies employ SFO directly for model parameter optimization. This observation suggests that the practical contribution of SFO in AI applications is primarily realized through optimization-assisted learning rather than replacing conventional training algorithms.

The temporal distribution of the studies also demonstrates an important evolution trend in the literature. Early studies mainly focused on standard SFO approaches for relatively direct optimization problems, while recent studies increasingly introduced hybrid and modified structures. This evolution indicates that researchers are attempting to adapt the algorithm to more complex and real-world optimization scenarios. Moreover, the increasing number of modified approaches shows that improving convergence behavior, search diversity, and optimization stability has become an important research direction in SFO-based studies.

Despite these strengths, several limitations can also be observed in the reviewed literature. One important limitation is that some research domains still contain a relatively limited number of studies. Compared to energy systems, communication networks, and image processing applications, fewer studies were identified in robotics, multi-objective optimization, and advanced industrial control systems. This distribution indicates that SFO still has significant research potential in several emerging application areas.

Another limitation observed in the literature is the increasing dependence on hybrid and modified structures to achieve higher optimization performance. Although hybridization improves solution quality and model performance in many applications, it also increases algorithmic complexity compared to the original SFO structure. In addition, similar to many population-based metaheuristic algorithms, SFO may experience premature convergence and become trapped in local optima when solving highly nonlinear or high-dimensional optimization problems. The optimization performance may also be sensitive to parameter settings, such as population size and the maximum number of iteration number, which can influence the balance between exploration and exploitation. These observations indicate that the standard SFO algorithm may require additional improvement strategies for more complex optimization problems. The growing number of modified and hybrid SFO variants in recent years further reflects ongoing efforts to improve search diversity, convergence behavior, and optimization stability while mitigating these limitations.

Although Scopus provides broad coverage of optimization, engineering, and artificial intelligence research, the use of a single database may be considered a methodological limitation. Some studies indexed only in Web of Science, IEEE Xplore, or Google Scholar may therefore not have been captured.

Another limitation that should be considered is the potential presence of publication bias in the reviewed literature. Studies reporting positive optimization results, improved performance, or successful applications are generally more likely to be published than studies reporting negative, neutral, or inconclusive findings. Consequently, the overall research landscape presented in this review may reflect a greater proportion of successful SFO applications. Therefore, the trends and conclusions reported in this review should be interpreted within the context of the available published literature.

From a practical perspective, the reviewed studies suggest that SFO has strong potential for intelligent optimization systems, particularly in smart grids, IoT systems, forecasting applications, image processing, and medical diagnosis frameworks. The increasing number of studies in these domains also indicates that SFO-based approaches are becoming more attractive for data-driven and intelligent engineering applications. In particular, the strong integration capability of SFO with machine learning and DL frameworks appears to be one of the major practical advantages of the algorithm.

Overall, the reviewed studies indicate that SFO has evolved into a flexible and effective optimization framework with strong hybridization capability and broad application diversity. At the same time, the literature highlights the need for further studies in underexplored application areas and the development of more efficient optimization structures for increasingly complex engineering and AI problems.

## 7. Open Challenges and Future Research Directions

The reviewed literature shows that SFO algorithm has experienced significant development in recent years through hybrid and modified optimization structures. However, several open challenges and future research opportunities still remain in the literature. The results presented in previous sections indicate that future SFO studies will likely focus on more adaptive, intelligent, and application-oriented optimization frameworks.

One of the major future research directions is the development of more efficient hybrid SFO frameworks. The reviewed studies clearly demonstrate that hybrid approaches have become dominant in many research domains, particularly in machine learning, image processing, IoT systems, and forecasting applications. This trend suggests that future studies may focus on improving the integration capability of SFO with AI models, DL frameworks, and other optimization algorithms. In particular, developing lighter and more adaptive hybrid structures may become an important research topic for handling increasingly complex optimization problems.

Another important research challenge is the application of SFO to real-time and large-scale optimization systems. The reviewed studies show that SFO has strong potential in energy systems, communication networks, and intelligent engineering applications. However, the increasing complexity of modern optimization problems requires optimization algorithms that can operate efficiently in dynamic and large-scale environments. Therefore, future studies may focus on developing SFO-based frameworks for real-time optimization, smart systems, and large-scale engineering applications.

The literature also indicates that some application domains remain relatively underexplored. Compared to energy systems, communication networks, and image processing applications, fewer studies were identified in robotics and multi-objective optimization problems. This situation suggests that future studies may further investigate the applicability of SFO in advanced robotics systems, industrial automation, constrained optimization, and multi-objective engineering problems.

Another promising research direction is the development of adaptive and self-improving SFO structures. The increasing number of modified SFO studies demonstrates that researchers are actively trying to improve convergence behavior, optimization stability, and search diversity. Future studies may therefore focus on adaptive parameter control strategies, dynamic search mechanisms, and self-adjusting optimization structures for improving the overall performance of the algorithm.

The strong integration tendency between SFO and AI systems also creates new research opportunities for intelligent optimization frameworks. The reviewed studies show that SFO has already been integrated with machine learning, feature selection, and prediction-oriented systems in many applications. Future studies may further investigate explainable, interpretable, and intelligent SFO-based optimization systems for data-driven engineering and AI applications.

Overall, the reviewed literature indicates that SFO still has considerable research potential in both theoretical and application-oriented optimization studies. The future development of SFO will likely depend on more adaptive hybrid frameworks, broader application diversity, and more intelligent optimization structures for solving increasingly complex engineering and AI problems.

## 8. Conclusions

This review study presented a comprehensive and systematic analysis of SFO algorithm and its application areas in engineering and AI research. The reviewed literature demonstrates that SFO has attracted increasing research interest in recent years and has been applied to a wide variety of optimization problems. The analysis performed in this study classified the existing literature into eight major research domains and evaluated the distribution of standard, hybrid, and modified SFO approaches across different application areas.

The reviewed studies show that SFO has been widely applied in engineering design, energy systems, image processing, communication networks, forecasting, machine learning, and intelligent optimization applications. This broad application diversity indicates that SFO can effectively handle nonlinear, multi-dimensional, and complex optimization problems in different research domains. In particular, the increasing number of studies in image processing, IoT systems, and prediction-oriented applications demonstrates the growing integration tendency between SFO and AI frameworks.

Another important finding of this review is the strong dominance of hybrid and modified SFO structures in recent years. The temporal and domain-based analyses reveal that researchers increasingly prefer hybrid optimization frameworks to improve convergence performance, optimization quality, and adaptability for more complex engineering problems. Especially in machine learning, forecasting, image processing, and communication systems, hybrid SFO approaches became more dominant than standard structures. In addition, the increasing number of modified SFO studies indicates continuous efforts to improve optimization stability, search diversity, and overall algorithmic performance.

The reviewed literature also demonstrates that some research domains still remain relatively underexplored. Compared to energy systems and communication-oriented applications, fewer studies were identified in robotics and multi-objective optimization problems. This situation suggests that SFO still has considerable research potential in emerging engineering and intelligent optimization applications.

Overall, the reviewed studies indicate that SFO has evolved from a relatively standard nature-inspired optimization algorithm into a flexible, adaptable, and hybridization-oriented optimization framework. The findings of this review suggest that SFO will likely continue attracting research interest in future intelligent engineering, machine learning, and complex optimization applications.

## Figures and Tables

**Table 1:** Classification of SFO applications in engineering design and structural optimization.

Ref.	Domain	Application	Variants of Sunflower (Standard/Hybrid/Modified)	Year
[23]	Structural Damage Identification	Damage identification in 3D truss structures	Hybrid	2023
[24]	Structural Damage Identification	Damage detection in laminated composites using mode shape curvature	Hybrid	2022
[25]	Structural Damage Identification	Damage identification in CFRP plates using strain fields	Standard	2021
[26]	Structural Damage Identification	Crack identification in laminated composites (ANN + RSM + SFO)	Hybrid	2021
[28]	Structural Damage Identification	Damage identification in composite structures (comparative study with SFO)	Standard	2020
[27]	Structural Damage Identification	Improved SFO tuning for structural damage identification	Modified	2021
[12]	Structural Damage Identification	Damage detection in laminated composite plates	Standard	2019
[29]	Composite Structural Design	Optimization of CFRP/GFRP composite tube design	Standard	2022
[30]	Composite Structural Design	Multi-objective design of CFRP isogrid structures	Hybrid	2021
[31]	Mechanical System Optimization	Airless tire design and multi-objective optimization	Hybrid	2025
[32]	Mechanical System Optimization	Design of quasi-zero stiffness vibration isolator	Standard	2025
[33]	Mechanical System Optimization	Structural optimization of Formula SAE chassis	Hybrid	2025
[34]	Mechanical System Optimization	Optimization of functionally graded plates	Standard	2023
[35]	Mechanical System Optimization	Optimization of shape memory origami structures	Hybrid	2025
[36]	Mechanical System Optimization	Efficiency optimization of mechanical reducer systems	Hybrid	2022
[37]	Manufacturing Process Optimization	Laser ablation process optimization	Hybrid	2024
[38]	Manufacturing Process Optimization	Electrochemical machining parameter optimization	Hybrid	2023
[39]	Manufacturing Process Optimization	Turning process optimization (Ti alloy machining)	Modified	2021
[40]	Manufacturing Process Optimization	Casting mould parameter optimization	Hybrid	2022
[41]	Manufacturing Process Optimization	FDM 3D printing parameter optimization	Hybrid	2022
[42]	Material & Construction Engineering	Optimization of bentonite-fly ash embankment core	Hybrid	2024
[43]	Material & Construction Engineering	Explainable AI-based concrete strength prediction and mix design	Modified	2023
[44]	Electrical Circuit Design	Fractional-order Butterworth filter design	Modified	2025

**Table 2:** Yearly distribution of SFO variants in engineering design and structural optimization.

Year	Standard	Hybrid	Modified	Total
2019	1	0	0	**1**
2020	1	0	0	**1**
2021	1	2	2	**5**
2022	1	4	0	**5**
2023	1	2	1	**4**
2024	0	2	0	**2**
2025	1	3	1	**5**
**Total**	**6**	**13**	**4**	**23**

**Table 3:** Classification of SFO applications in energy systems and power engineering.

Ref.	Domain	Application	Variants of Sunflower (Standard/Hybrid/Modified)	Year
[13]	Battery Modeling, Parameter Identification, and State Estimation (SoC/Capacity)	Battery parameter identification & SoC estimation	Standard	2025
[14]	Battery Modeling, Parameter Identification, and State Estimation (SoC/Capacity)	SOC estimation using optimized EKF	Hybrid	2022
[15]	Battery Modeling, Parameter Identification, and State Estimation (SoC/Capacity)	Battery capacity estimation	Standard	2022
[47]	Battery Modeling, Parameter Identification, and State Estimation (SoC/Capacity)	Online battery parameter estimation	Standard	2021
[46]	Battery Modeling, Parameter Identification, and State Estimation (SoC/Capacity)	Real-time battery parameter identification	Standard	2020
[45]	Battery Modeling, Parameter Identification, and State Estimation (SoC/Capacity)	SoC estimation & parameter identification	Modified	2020
[18]	PV Systems, Fuel Cells, and Power Converter Modeling and Optimization	PV inverter/DC-AC converter optimization	Standard	2024
[16]	PV Systems, Fuel Cells, and Power Converter Modeling and Optimization	Solar cell parameter identification	Modified	2023
[19]	PV Systems, Fuel Cells, and Power Converter Modeling and Optimization	MPPT & reactive power control in PV	Hybrid	2023
[20]	PV Systems, Fuel Cells, and Power Converter Modeling and Optimization	PEM fuel cell parameter estimation	Modified	2020
[17]	PV Systems, Fuel Cells, and Power Converter Modeling and Optimization	Three-diode PV model identification	Hybrid	2019
[21]	Techno-Economic Design and Optimization of Hybrid Energy Systems (CCHP/micro-CHP)	LNG-based CCHP system optimization	Hybrid	2025
[22]	Techno-Economic Design and Optimization of Hybrid Energy Systems (CCHP/micro-CHP)	PEMFC-based CCHP design optimization	Modified	2024
[48]	Techno-Economic Design and Optimization of Hybrid Energy Systems (CCHP/micro-CHP)	Heat pump selection in micro-CHP	Standard	2020
[49]	Techno-Economic Design and Optimization of Hybrid Energy Systems (CCHP/micro-CHP)	PV-Fuel cell hybrid system optimization	Modified	2022
[54]	Energy Management and Scheduling in Smart Grids and Microgrids	Two-stage energy scheduling	Modified	2025
[53]	Energy Management and Scheduling in Smart Grids and Microgrids	Smart home energy management	Hybrid	2025
[52]	Energy Management and Scheduling in Smart Grids and Microgrids	CHHP microgrid energy management	Hybrid	2025
[51]	Energy Management and Scheduling in Smart Grids and Microgrids	Multi-stage energy system optimization	Modified	2024
[50]	Energy Management and Scheduling in Smart Grids and Microgrids	Day-ahead grid scheduling	Modified	2024
[58]	Energy Management and Scheduling in Smart Grids and Microgrids	EV-based microgrid scheduling	Hybrid	2024
[57]	Energy Management and Scheduling in Smart Grids and Microgrids	Joint EV + DR scheduling	Modified	2024
[56]	Energy Management and Scheduling in Smart Grids and Microgrids	Microgrid generation scheduling	Modified	2024
[55]	Energy Management and Scheduling in Smart Grids and Microgrids	Economic scheduling with EVs	Modified	2024
[61]	Controller Design, Frequency Regulation, and Stability Enhancement in Power Systems	Frequency control (FOFPID)	Standard	2025
[63]	Controller Design, Frequency Regulation, and Stability Enhancement in Power Systems	PI controller optimization	Standard	2024
[62]	Controller Design, Frequency Regulation, and Stability Enhancement in Power Systems	Adaptive PI control	Hybrid	2022
[64]	Controller Design, Frequency Regulation, and Stability Enhancement in Power Systems	Microgrid control	Standard	2022
[60]	Controller Design, Frequency Regulation, and Stability Enhancement in Power Systems	AGC with fuel cell	Standard	2022
[59]	Controller Design, Frequency Regulation, and Stability Enhancement in Power Systems	Frequency regulation (hybrid system)	Modified	2021
[65]	Controller Design, Frequency Regulation, and Stability Enhancement in Power Systems	Power system stabilizer tuning	Modified	2020
[66]	Controller Design, Frequency Regulation, and Stability Enhancement in Power Systems	Automatic generation control	Standard	2020
[72]	Distribution Network Reconfiguration, Topology Optimization, and Distributed Generation Placement	Network reconfiguration + DG placement	Modified	2022
[71]	Distribution Network Reconfiguration, Topology Optimization, and Distributed Generation Placement	DG placement	Standard	2022
[73]	Distribution Network Reconfiguration, Topology Optimization, and Distributed Generation Placement	Network topology optimization	Standard	2021
[69]	Distribution Network Reconfiguration, Topology Optimization, and Distributed Generation Placement	Network reconfiguration	Modified	2021
[75]	Distribution Network Reconfiguration, Topology Optimization, and Distributed Generation Placement	DG location optimization	Standard	2021
[74]	Distribution Network Reconfiguration, Topology Optimization, and Distributed Generation Placement	Network reconfiguration	Standard	2021
[70]	Distribution Network Reconfiguration, Topology Optimization, and Distributed Generation Placement	DG placement	Modified	2021
[76]	Distribution Network Reconfiguration, Topology Optimization, and Distributed Generation Placement	Distribution efficiency optimization	Modified	2020
[68]	Distribution Network Reconfiguration, Topology Optimization, and Distributed Generation Placement	Techno-economic network reconfiguration	Standard	2021
[67]	Distribution Network Reconfiguration, Topology Optimization, and Distributed Generation Placement	Optimal network topology	Standard	2021
[83]	OPF, Reactive Power Dispatch, and Economic-Emission Load Dispatch	Economic-emission dispatch	Modified	2022
[79]	OPF, Reactive Power Dispatch, and Economic-Emission Load Dispatch	OPF	Standard	2021
[81]	OPF, Reactive Power Dispatch, and Economic-Emission Load Dispatch	Reactive power dispatch	Standard	2021
[80]	OPF, Reactive Power Dispatch, and Economic-Emission Load Dispatch	Reactive power generation	Standard	2021
[82]	OPF, Reactive Power Dispatch, and Economic-Emission Load Dispatch	OPF (hybrid method)	Hybrid	2020
[78]	OPF, Reactive Power Dispatch, and Economic-Emission Load Dispatch	SCOPF	Standard	2020
[77]	OPF, Reactive Power Dispatch, and Economic-Emission Load Dispatch	OPF with DG	Standard	2019

**Table 4:** Yearly distribution of SFO variants in energy systems and power engineering studies.

Year	Standard	Hybrid	Modified	Total
2019	1	1	0	**2**
2020	4	1	4	**9**
2021	9	0	3	**12**
2022	4	2	3	**9**
2023	0	1	1	**2**
2024	2	1	6	**9**
2025	2	3	1	**6**
**Total**	**22**	**9**	**18**	**49**

**Table 5:** Classification of SFO applications in machine learning and feature selection.

Ref.	Domain	Application	Variants of Sunflower (Standard/Hybrid/Modified)	Year
[84]	Biomedical and Healthcare Machine Learning Applications	Transcriptomic biomarker recognition using SFO-RFE	Hybrid	2024
[85]	DL and NLP-Based Intelligent Systems	Feature selection using chaotic and Lévy-flight binary SFO	Hybrid	2025
[86]	Explainable AI and Interpretable Machine Learning	Diabetes diagnosis using SFO-optimized ML and SHAP	Modified	2026
[87]	Explainable AI and Interpretable Machine Learning	Parkinson’s disease classification using CSSFOA-optimized ZF-Net	Standard	2025
[88]	Agriculture and Smart Environment Applications	Textual emotion recognition using attention-based LSTM and chaotic SFO	Hybrid	2026
[89]	Agriculture and Smart Environment Applications	XAI-based food drying performance analysis using OCSFO	Hybrid	2024
[90]	Biomedical and Healthcare Machine Learning Applications	Explainable analysis of bovine uterine gas data using SFO	Hybrid	2024
[91]	DL and NLP-Based Intelligent Systems	Seed germination prediction using crossover-boosted SFO	Hybrid	2025
[92]	Explainable AI and Interpretable Machine Learning	Smart irrigation control using Deep CNN optimized by SFO	Modified	2026

**Table 6:** Yearly distribution of SFO variants in machine learning and feature selection studies.

Year	Standard	Hybrid	Modified	Total
2024	0	3	0	**3**
2025	1	2	0	**3**
2026	0	1	2	**3**
**Total**	**1**	**6**	**2**	**9**

**Table 7:** Classification of SFO applications in image processing and medical applications.

Ref.	Domain	Application	Variants of Sunflower (Standard/Hybrid/Modified)	Year
[93]	Medical Image Analysis, Disease Diagnosis, and Clinical Decision Support	Oral cancer diagnosis using deep transfer learning	Hybrid	2026
[95]	Medical Image Analysis, Disease Diagnosis, and Clinical Decision Support	Brain tumor classification using optimized CNN	Hybrid	2024
[97]	Medical Image Analysis, Disease Diagnosis, and Clinical Decision Support	Parkinson’s disease classification using CNN + BiLSTM	Hybrid	2024
[99]	Medical Image Analysis, Disease Diagnosis, and Clinical Decision Support	Lung nodule detection and classification	Modified	2023
[100]	Medical Image Analysis, Disease Diagnosis, and Clinical Decision Support	Heart disease prediction using CNN	Hybrid	2023
[98]	Medical Image Analysis, Disease Diagnosis, and Clinical Decision Support	Lung cancer diagnosis using CT images	Hybrid	2023
[96]	Medical Image Analysis, Disease Diagnosis, and Clinical Decision Support	Alzheimer’s and Parkinson’s diagnosis using KELM	Standard	2022
[94]	Medical Image Analysis, Disease Diagnosis, and Clinical Decision Support	Brain tumor segmentation using FrCN	Modified	2022
[102]	Medical Image Analysis, Disease Diagnosis, and Clinical Decision Support	Mammogram classification using ELM	Modified	2021
[103]	Medical Image Analysis, Disease Diagnosis, and Clinical Decision Support	Liver cancer biomarker-based diagnosis	Hybrid	2020
[101]	Medical Image Analysis, Disease Diagnosis, and Clinical Decision Support	Breast cancer diagnosis using DBN	Modified	2020
[104]	Medical Image Compression and Reconstruction	MRI image compression and reconstruction	Hybrid	2023
[105]	Medical Image Compression and Reconstruction	Compressive sensing-based image compression	Modified	2023
[106]	Agricultural and Plant Disease Image Analysis	Pomegranate disease detection using DL	Hybrid	2025
[108]	Agricultural and Plant Disease Image Analysis	Paddy leaf disease classification using SVM	Modified	2025
[109]	Agricultural and Plant Disease Image Analysis	Crop disease detection using DL	Standard	2024
[107]	Agricultural and Plant Disease Image Analysis	Paddy blast disease prediction	Modified	2024
[110]	Agricultural and Plant Disease Image Analysis	Rice leaf disease classification (IoT + DL)	Hybrid	2023
[111]	Agricultural and Plant Disease Image Analysis	Groundnut disease detection using ML	Hybrid	2023
[112]	Agricultural and Plant Disease Image Analysis	Tomato leaf disease segmentation	Modified	2022
[113]	Remote Sensing and Geospatial Image Analysis	Remote sensing image retrieval using CNN	Modified	2025
[114]	Remote Sensing and Geospatial Image Analysis	Land cover classification using DBN	Hybrid	2023
[115]	Image Retrieval, General Image Classification, and Vision-Based Recognition	Human action recognition in video	Hybrid	2022
[116]	Image Retrieval, General Image Classification, and Vision-Based Recognition	Content-based image retrieval (CBIR)	Modified	2022
[117]	Image Retrieval, General Image Classification, and Vision-Based Recognition	License plate recognition	Hybrid	2022
[118]	Image Retrieval, General Image Classification, and Vision-Based Recognition	General image classification using NN	Hybrid	2021
[119]	Image Enhancement, Restoration, Compression, and Multimedia Security	Video forgery detection	Hybrid	2025
[120]	Image Enhancement, Restoration, Compression, and Multimedia Security	Multispectral image denoising	Standard	2025
[121]	Image Enhancement, Restoration, Compression, and Multimedia Security	Compressed sensing for image/video reconstruction	Hybrid	2024
[122]	Image Enhancement, Restoration, Compression, and Multimedia Security	Digital image watermarking	Hybrid	2023
[123]	Image Enhancement, Restoration, Compression, and Multimedia Security	Image encryption using chaotic hybrid SFO	Hybrid	2022

**Table 8:** Yearly distribution of SFO variants in image processing and medical applications studies.

Year	Standard	Hybrid	Modified	Total
2020	0	1	1	**2**
2021	0	1	1	**2**
2022	1	3	3	**7**
2023	0	7	2	**9**
2024	1	3	1	**5**
2025	1	2	2	**5**
2026	0	1	0	**1**
**Total**	**3**	**18**	**10**	**31**

**Table 9:** Classification of SFO applications in communication networks and IoT systems.

Ref.	Domain	Application	Variants of Sunflower (Standard/Hybrid/Modified)	Year
[132]	Energy-Efficient Clustering, CH Selection, Network Lifetime Optimization	CH selection in WSN-IoT	Modified	2026
[128]	Energy-Efficient Clustering, CH Selection, Network Lifetime Optimization	Secure clustering and transmission	Modified	2024
[131]	Energy-Efficient Clustering, CH Selection, Network Lifetime Optimization	LEACH-based energy-efficient clustering	Modified	2023
[127]	Energy-Efficient Clustering, CH Selection, Network Lifetime Optimization	K-medoids clustering and routing	Hybrid	2023
[129]	Energy-Efficient Clustering, CH Selection, Network Lifetime Optimization	Network lifetime improvement using hybrid optimization	Hybrid	2023
[126]	Energy-Efficient Clustering, CH Selection, Network Lifetime Optimization	Grey wolf–sunflower hybrid CH selection	Hybrid	2021
[125]	Energy-Efficient Clustering, CH Selection, Network Lifetime Optimization	Improved SFO for CH selection	Modified	2021
[130]	Energy-Efficient Clustering, CH Selection, Network Lifetime Optimization	Cross-layer routing with coverage optimization	Modified	2020
[124]	Energy-Efficient Clustering, CH Selection, Network Lifetime Optimization	Modified SFO for WSN lifetime maximization	Modified	2020
[134]	Routing, Data Collection, Data Aggregation, Mobile Sink Optimization	Mobile data collection scheduling (IoT-WSN)	Hybrid	2026
[136]	Routing, Data Collection, Data Aggregation, Mobile Sink Optimization	Cross-layer routing optimization	Hybrid	2025
[138]	Routing, Data Collection, Data Aggregation, Mobile Sink Optimization	Data aggregation using DE + SFO	Hybrid	2024
[137]	Routing, Data Collection, Data Aggregation, Mobile Sink Optimization	Cluster-based data aggregation	Modified	2023
[135]	Routing, Data Collection, Data Aggregation, Mobile Sink Optimization	Opportunistic routing and denoising	Hybrid	2021
[133]	Routing, Data Collection, Data Aggregation, Mobile Sink Optimization	Mobile sink routing optimization	Hybrid	2020
[144]	Intrusion Detection, Attack Detection, Secure Communication	Secure routing in MANET-IoT healthcare	Hybrid	2025
[139]	Intrusion Detection, Attack Detection, Secure Communication	APT attack detection using DL	Hybrid	2025
[148]	Intrusion Detection, Attack Detection, Secure Communication	Cyber threat detection with DL	Standard	2025
[143]	Intrusion Detection, Attack Detection, Secure Communication	Intrusion detection + routing optimization	Standard	2024
[142]	Intrusion Detection, Attack Detection, Secure Communication	DoS attack detection in WSN	Modified	2024
[141]	Intrusion Detection, Attack Detection, Secure Communication	Intrusion detection with optimal routing	Standard	2024
[140]	Intrusion Detection, Attack Detection, Secure Communication	DDoS attack detection with CNN	Standard	2023
[147]	Intrusion Detection, Attack Detection, Secure Communication	IIoT intrusion detection	Standard	2023
[146]	Intrusion Detection, Attack Detection, Secure Communication	Trust-based routing optimization	Hybrid	2021
[145]	Intrusion Detection, Attack Detection, Secure Communication	Trust-aware routing with autoencoder	Hybrid	2021
[150]	Blockchain & Cryptography-Based Privacy and Security	Blockchain-based healthcare privacy	Hybrid	2025
[152]	Blockchain & Cryptography-Based Privacy and Security	Secure cloud auditing with encryption	Hybrid	2024
[149]	Blockchain & Cryptography-Based Privacy and Security	Blockchain-based IoT healthcare security	Modified	2024
[151]	Blockchain & Cryptography-Based Privacy and Security	Secure cloud encryption and auditing	Modified	2023
[154]	Blockchain & Cryptography-Based Privacy and Security	VANET privacy-preserving cryptography	Standard	2022
[153]	Blockchain & Cryptography-Based Privacy and Security	Blockchain-based secure routing (VANET)	Hybrid	2022
[155]	Cloud Computing, Task Scheduling, Load Balancing	Cloud load balancing	Hybrid	2022
[156]	Cloud Computing, Task Scheduling, Load Balancing	Data transfer scheduling in cloud	Modified	2023
[157]	Cloud Computing, Task Scheduling, Load Balancing	Cloud-based intrusion detection	Modified	2023
[158]	Cloud Computing, Task Scheduling, Load Balancing	Resource allocation in cloud	Hybrid	2021
[160]	Wireless Communication System Optimization	Antenna selection in Massive MIMO	Hybrid	2023
[159]	Wireless Communication System Optimization	Energy-efficient antenna selection	Hybrid	2023
[161]	Wireless Communication System Optimization	Spectrum sensing and PAPR reduction	Hybrid	2022
[162]	Wireless Communication System Optimization	Controller placement in SD-IoT	Modified	2022
[165]	IoT-Enabled Smart Agriculture and Plant Monitoring	Secure routing + plant disease prediction	Hybrid	2023
[164]	IoT-Enabled Smart Agriculture and Plant Monitoring	Intrusion detection in smart agriculture	Hybrid	2023
[163]	IoT-Enabled Smart Agriculture and Plant Monitoring	Routing + disease prediction	Hybrid	2021
[166]	Vehicular Ad Hoc Networks and Intelligent Transportation	Driver behavior prediction in VANET	Hybrid	2021

**Table 10:** Yearly distribution of SFO variants in communication networks and IoT systems studies.

Year	Standard	Hybrid	Modified	Total
2020	0	1	2	**3**
2021	0	7	1	**8**
2022	1	3	1	**5**
2023	2	6	5	**13**
2024	2	2	3	**7**
2025	1	4	0	**5**
2026	0	1	1	**2**
**Total**	**6**	**24**	**13**	**43**

**Table 11:** Classification of SFO applications in control systems and robotics.

Ref.	Domain	Application	Variants of Sunflower (Standard/Hybrid/Modified)	Year
[167]	Radar, Signal Processing, and Estimation Systems	Target number and angle-range estimation in FDA-MIMO radar	Modified	2025
[168]	Electric Vehicle and Motor Control Systems	EV speed tracking using FOPD controller	Standard	2024
[169]	Electric Vehicle and Motor Control Systems	SRM speed and current control with torque ripple minimization	Hybrid	2022
[170]	Electric Vehicle and Motor Control Systems	Parameter estimation of six-phase induction motor	Standard	2022
[171]	Power Systems and Microgrid Control	Frequency regulation in hybrid distributed power systems	Modified	2021
[172]	Power Systems and Microgrid Control	Optimal PI control for microgrid operation	Standard	2021
[173]	Power Systems and Microgrid Control	Adaptive PI controller for microgrid stability	Hybrid	2021
[174]	Robotic Motion Planning and Navigation	Motion and orientation control of humanoid robot (NAO)	Standard	2021
[175]	Robotic Motion Planning and Navigation	Motion planning of wheeled robot (Pioneer P3-DX)	Standard	2021
[176]	Multi-Robot Systems and Advanced Robotic Optimization	Multi-humanoid robot trajectory planning	Hybrid	2022

**Table 12:** Yearly distribution of SFO variants in control systems and robotics studies.

Year	Standard	Hybrid	Modified	Total
2021	3	1	1	**5**
2022	1	2	0	**3**
2024	1	0	0	**1**
2025	0	0	1	**1**
**Total**	**5**	**3**	**2**	**10**

**Table 13:** Classification of SFO applications in data analytics, forecasting and prediction.

Ref.	Domain	Application	Variants of Sunflower (Standard/Hybrid/Modified)	Year
[178]	Environmental, Hydrological and Climate Forecasting	Runoff/streamflow prediction (SWAT-MLP)	Modified	2024
[179]	Environmental, Hydrological and Climate Forecasting	Pan evaporation prediction (MLP-based hybrid models)	Hybrid	2022
[177]	Environmental, Hydrological and Climate Forecasting	Streamflow forecasting using MLP-SFO	Hybrid	2021
[180]	Environmental, Hydrological and Climate Forecasting	Soil temperature prediction using hybrid ML models	Hybrid	2021
[181]	Environmental, Hydrological and Climate Forecasting	Lake water level prediction using ANFIS-SFO	Hybrid	2021
[182]	Energy Systems, Industrial Process and Materials Prediction	CO/CO_2_ prediction in gasification process	Hybrid	2025
[183]	Energy Systems, Industrial Process and Materials Prediction	Electrochemical property prediction of biomass materials	Hybrid	2025
[184]	Energy Systems, Industrial Process and Materials Prediction	Electricity demand forecasting	Hybrid	2023
[185]	Energy Systems, Industrial Process and Materials Prediction	Ethylene production prediction using LSTM-SFO	Hybrid	2022
[186]	Disaster, Hazard and Risk Prediction	Seismic hazard prediction in mining	Hybrid	2025
[187]	Disaster, Hazard and Risk Prediction	Flood and earthquake detection using DL	Hybrid	2023
[188]	Civil, Geotechnical and Transportation Prediction	Pile settlement prediction using MLP	Hybrid	2024
[189]	Civil, Geotechnical and Transportation Prediction	Traffic flow prediction using cellular automata	Modified	2021
[190]	Financial and Business Intelligence Prediction	Financial crisis prediction (ML model tuning)	Hybrid	2024
[191]	Financial and Business Intelligence Prediction	Customer churn prediction using DL + optimization	Hybrid	2021
[192]	Text Analytics, Summarization and Information Retrieval	Multi-document summarization using GAN & RNN	Hybrid	2021
[193]	Text Analytics, Summarization and Information Retrieval	Cross-lingual summarization and query optimization	Hybrid	2023
[194]	Sentiment Analysis and Social Media Analytics	Sentiment analysis using BiLSTM	Hybrid	2023
[195]	Sentiment Analysis and Social Media Analytics	COVID-19 tweet sentiment analysis	Hybrid	2022
[197]	Medical Data Analytics and Clustering	Medical data clustering using RSFO	Hybrid	2022
[196]	Medical Data Analytics and Clustering	K-means based hybrid clustering	Hybrid	2022
[198]	Marketing, Advertising and User-Oriented Predictive Analytics	Location-based advertising prediction	Hybrid	2022
[199]	Computational Intelligence Infrastructure and Scheduling Analytics	Cloud task scheduling optimization	Modified	2022
[200]	Predictive Classification in Applied Data Mining	Biodegradable classification using SFO-TLBO	Hybrid	2021

**Table 14:** Yearly distribution of SFO variants in data analytics, forecasting and prediction studies.

Year	Standard	Hybrid	Modified	Total
2021	0	6	1	**7**
2022	0	6	1	**7**
2023	0	4	0	**4**
2024	0	2	1	**3**
2025	0	3	0	**3**
**Total**	**0**	**21**	**3**	**24**

**Table 15:** Classification of SFO applications in multi-objective and benchmark optimization.

Ref.	Domain	Application	Variants of Sunflower (Standard/Hybrid/Modified)	**Year**
[201]	Hybrid and Improved Metaheuristic Algorithm Design	Development of Hybrid Butter-Flower Algorithm for global optimization	Hybrid	2025
[202]	Multi-Objective Optimization and Pareto-Based Approaches	Development of MOSFO for constrained multi-objective optimization and Pareto front generation	Modified	2023
[203]	Benchmark-Based Validation in Applied Optimization Frameworks	Cyber forensic framework using SFO-Jaya optimized deep stacked autoencoder	Hybrid	2021

**Table 16:** Yearly distribution of SFO variants in multi-objective and benchmark optimization.

Year	Standard	Hybrid	Modified	Total
2021	0	1	0	**1**
2023	0	0	1	**1**
2025	0	1	0	**1**
**Total**	**0**	**2**	**1**	**3**

**Table 17:** Research domain-based distribution of SFO studies.

Domain	Standard	Hybrid	Modified	Total
Engineering Design and Structural Optimization	6	13	4	**23**
Energy Systems and Power Engineering	22	9	18	**49**
Machine Learning and Feature Selection	1	6	2	**9**
Image Processing and Medical Applications	3	18	10	**31**
Communication Networks and IoT Systems	6	24	13	**43**
Control Systems and Robotics	5	3	2	**10**
Data Analytics, Forecasting and Prediction	0	21	3	**24**
Multi-Objective and Benchmark Optimization	0	2	1	**3**

## Data Availability

No new data were created or analyzed in this study. Data sharing is not applicable.

## Abbreviations

The following abbreviations are used in this manuscript:

**Abbreviation**	**Explanation**
AI	Artificial intelligence
ANFIS	Adaptive neuro-fuzzy inference system
ANN	Artificial neural network
APT	Advanced persistent threat
BiLSTM	Bidirectional long short-term memory
CCHP	Combined cooling, heating, and power
CH	Cluster head
CNN	Convolutional neural network
DE	Differential evolution
DG	Distribution generation
DL	Deep learning
EKF	Extended Kalman filter
FDA	Frequency diverse array
GA	Genetic Algorithm
GAN	Generative adversarial network
GWO	Grey wolf optimizer
IIoT	Industrial internet of things
IoT	Internet of things
LSTM	Long short-term memory
MANET	Mobile ad hoc networks
MIMO	Multiple input multiple output
MPPT	Maximum power point tracking
NLP	Natural language processing
OPF	Optimal power flow
PFA	Polar Fox Optimization Algorithm
PI	Proportional–integral
PID	Proportional integral derivative
PSO	Particle Swarm Optimization
PV	Photovoltaic
RFE	Recursive feature elimination
RNN	Recurrent neural network
RSM	Response surface method
SAR	Synthetic-aperture radar
SD	Software defined
SDN	Software defined networking
SFO	Sunflower optimization algorithm
SHM	Structural health monitoring
SoC	State of charge
SRM	Switched reluctance motor
VANET	Vehicular ad hoc network
WSN	Wireless sensor network
